# Deciphering the Tumor Uptake of Heterobivalent (SST_2_/Albumin) [^64^Cu]Cu-NODAGA-cLAB-TATEs

**DOI:** 10.1021/acs.jmedchem.5c00890

**Published:** 2025-05-20

**Authors:** Florian Brandt, Martin Ullrich, Markus Laube, Reik Löser, Jörg Kotzerke, Klaus Kopka, Jens Pietzsch, Jörg van den Hoff, Robert Wodtke

**Affiliations:** † 28414 Helmholtz-Zentrum Dresden-Rossendorf, Institute of Radiopharmaceutical Cancer Research, Bautzner Landstraße 400, Dresden 01328, Germany; ‡ University Hospital Carl Gustav Carus at the Technische Universität Dresden, Klinik und Poliklinik für Nuklearmedizin, Fetscherstraße 74, Dresden 01307, Germany; § Technische Universität Dresden, Faculty of Chemistry and Food Chemistry, Mommsenstraße 4, Dresden 01069, Germany; ∥ German Cancer Consortium (DKTK), Partner Site Dresden, Dresden 01307, Germany; ⊥ National Center for Tumor Diseases (NCT), Partner Site Dresden, University Cancer Center (UCC), Dresden 01307, Germany

## Abstract

Radioligands with albumin-binding moieties exhibit a great potential for the treatment of tumor diseases owing to the general finding of an increased integral tumor uptake compared to radioligands without such moieties. However, the reasons for this pharmacokinetic behavior are less explored. Herein, we focused on identifying potential mechanisms for our previously developed heterobivalent (SST_2_/albumin) **[** ^**64**^ **Cu]­Cu-NODAGA-cLAB-TATEs**. For this purpose, we designed two novel derivatives that show either negligible binding to albumin or lack the SST_2_-targeting capability. Based on the *in vivo* results, we hypothesize that binding of the albumin-bound radioligand to SST_2_ in addition to that of the free radioligand causes the increased tumor uptake. This is supported by saturation binding analyses in the presence of albumin and compartment modeling considerations. Overall, the results of this study provide a first tentative explanation for the phenomenon of increased tumor uptake for albumin-binding radioligands, which may support the prospective design of such radioligands.

## Graphical abstract

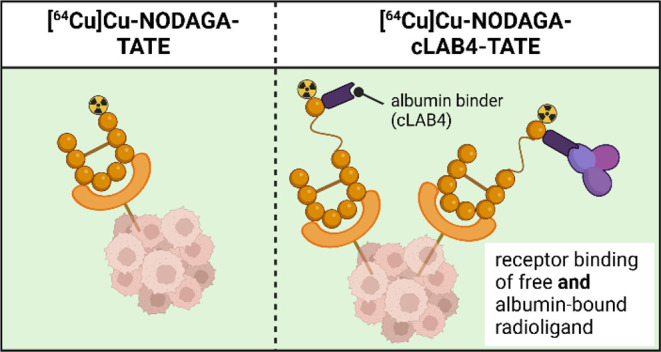

## Introduction

The modification of radioligands for targeted radionuclide therapy with albumin-binding moieties (or albumin binders) has been proposed as a means to increase the integral tumor uptake. The first report on the favorable impact of albumin binders on tumor uptake of targeted radiopharmaceuticals was presented by Cristina Müller and colleagues in 2013 for a folate receptor ligand.^1^ The low-molecular-weight albumin binder Lys­(4-(*p*-iodophenyl)­butanoyl)–OH (Lys­(IPB)–OH) was used for functionalization based on the original discovery of Dumelin et al.^2^ in 2008 showing that this chemotype exhibits a binding affinity to albumin in the low μM range. Since these pioneering studies, albumin-binding radioligands for various targets were developed, exploiting either Lys­(IPB) derivatives or a truncated Evans Blue analog as the albumin-binding moiety.^3,4^ Moreover, Wen et al.^5^ recently reported on the use of Lys­(Dansyl-Phe) as another albumin-binding chemotype. The principal finding of these studies is, that the integral tumor uptake is higher for these radioligands than for the respective radioligands without an albumin binder. This prompted the translation of radioligands targeting PSMA,^6–9^ SST_2_ ^10–13^ and FAP^14^ to first in-human studies. However, the improved integral tumor uptake is accompanied by an increased retention (prolonged circulation) of radioactivity in the blood pool. Thus, increased hematoxicity is a potential concern for radionuclide therapeutic applications with albumin-binding radioligands.

Although albumin binders have been successfully applied to increase the integral tumor uptake, the particular mechanisms that lead to this increase are poorly understood. Generally, binding to albumin should not increase the integral tumor uptake *per se* as the total area under the concentration–time curve (AUC) of the free (unbound) radioligand fraction, which initially should be assumed to be the sole target-reactive species, is not altered by albumin binding.^15–17^ Accordingly, the common statement that the increase in blood circulation time of the radioligand due to binding to albumin leads to an increased tumor uptake is erroneous. An increased total dose delivery to the tumor must thus be caused by an additional or modified tumor uptake mechanism such as target binding of the albumin-bound radioligand and/or a favorable structural influence of the albumin-binding moiety on the tumor targeting that is independent of the albumin-binding ability. The latter aspect could also mean that the albumin-binding moiety itself addresses a distinct target at the tumor cells. Moreover, the albumin-bound radioligand can be retained in the tumor tissue by additional uptake mechanisms that do not rely on the actual molecular target and its vector molecule but rather on albumin itself (i.e., enhanced permeability and retention effect, EPR).^18^ The contributions of the aforementioned mechanisms that might cause an increased integral tumor uptake certainly depend on the particular target, the albumin-binding moiety, and tumor entity, and a better mechanistic understanding might support the clinical translation of albumin-binding radioligands.

Previously, we developed “clickable” lysine-derived albumin binders (cLABs) bearing azide or alkyne moieties that enable the late-stage modification of target molecules with the opposing functionality via copper-catalyzed azide–alkyne cycloaddition (CuAAC).^19^ From a small library of these cLABs, four azide-functionalized cLABs were selected for coupling to the somatostatin-receptor subtype-2 (SST_2_) agonist (Tyr^3^) octreotate (TATE), which was modified with a short PEG2 linker and a propargylglycine residue. The resulting peptide conjugates, named **NODAGA-cLAB­(1–4)-TATEs** (Figure 1, with NODAGA being the radiometal-chelating unit), were labeled with the positron-emitting radionuclide copper-64. We demonstrated their heterobivalence, i.e., binding to SST_2_ and albumin, in *in vitro* and *in vivo* studies. The albumin-binding affinity of the **[** ^**64**^ **Cu]­Cu-NODAGA-cLAB­(1–4)-TATEs** ranged from 1.8 to 50 μM. We showed that the tumor uptake of these heterobivalent radioligands is protracted compared to TATE derivatives without a distinct albumin-binding moiety and that this protraction increased with increasing binding affinity to albumin. Among these radioligands, **[** ^**64**^ **Cu]­Cu-NODAGA-cLAB4-TATE** for which N_3_Bz-l-Lys­(4-(*p*-methylphenyl)­butanoyl)-NH_2_ was used as **cLAB**, exhibited an albumin-binding affinity of 50 μM and showed the best *in vivo* performance regarding integral tumor uptake over 48 h and tumor-to-kidney ratio. It is worth noting that both metrics are improved in comparison to **[** ^**64**^ **Cu]­Cu-NODAGA-TATE**.^19^ In line with these results, **[** ^**67**^ **Cu]­Cu-NODAGA-cLAB4-TATE** proved to be suitable for a therapeutic approach toward neuroendocrine tumors as demonstrated by us recently in an animal model.^20^

**Figure 1:**
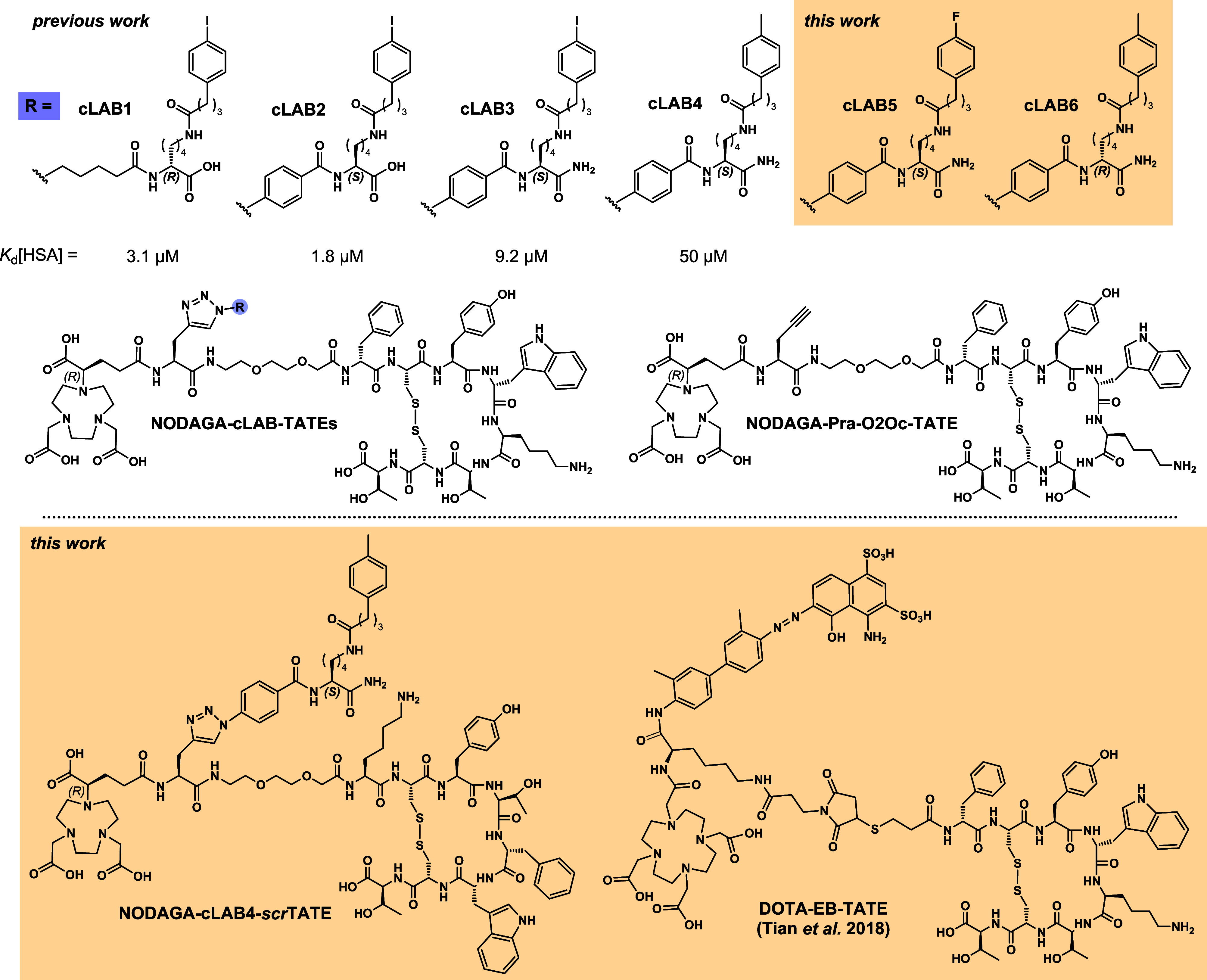
Structures of TATE derivatives characterized previously and herein. The amino acid sequence of TATE is d-Phe-c­(Cys-Tyr-d-Trp-Lys-Thr-Cys)-Thr and that of *scr*TATE (scrambled TATE) is Lys-c­(Cys-Tyr-Thr-d-Phe-d-Trp-Cys)-Thr.

The present study continues our previous work with the primary focus on deciphering the reasons for the increased integral tumor uptake of **[** ^**64**^ **Cu]­Cu-NODAGA-cLAB4-TATE** compared to **[** ^**64**^ **Cu]­Cu-NODAGA-TATE**. For this purpose, we sought to assess the tumor uptake of the free, i.e., albumin-unbound, and albumin-bound fraction and designed two novel compounds accordingly. For **NODAGA-cLAB5-TATE** (Figure 1), 4-N_3_Bz-l-Lys­(4-(*p*-fluorophenyl)­butanoyl)-NH_2_ was chosen as **cLAB** that should exhibit a negligible binding affinity to albumin based on our previous structure–activity relationships.^19^ Therefore, this compound might give insight into the structural implications of the **cLABs** on the *in vivo* targeting of SST_2_ apart from their albumin-binding ability. On the other hand, **NODAGA-cLAB4-** *scr* **TATE** (Figure 1) bears the same albumin-binding moiety as **NODAGA-cLAB4-TATE**, but the SST_2_ targeting unit TATE was replaced by a scrambled TATE (*scr*TATE). For *scr*TATE, the positions of d-Trp^4^ and Lys^5^ were swapped with Thr^6^ and d-Phe^1^, respectively, while the positions of the two Cys residues were kept constant to maintain the same size of the cyclic structure as found in the original TATE. These structural modifications should prevent SST_2_ binding and residual tumor uptake (if at all) might then originate from the albumin-bound radioligand and/or off-target binding by the albumin-binding moiety itself.

In addition to these two compounds, we synthesized **NODAGA-cLAB6-TATE** (Figure 1) for which 4-N_3_Bz-d-Lys­(4-(*p*-methylphenyl)­butanoyl)-NH_2_ was used as **cLAB**, which is the d-configured pendant to **cLAB4**. Previously, we noted that the lysine amide functionality is hydrolyzed in mouse plasma to the respective carboxylic acid by plasma carboxylesterases.^19^ As this transformation entails also an increase in the binding affinity to albumin, we were interested in studying the time profile of tumor uptake without the occurrence of this transformation. We complement our investigations of the **NODAGA-cLAB-TATEs** by comparing their tissue distribution profile and time-dependent tumor uptake with another known albumin-binding SST_2_ ligand. **DOTA-EB-TATE** bears a truncated Evans Blue derivative as albumin-binding moiety and the promising preclinical results for ^86^Y-, ^90^Y- and ^177^Lu-labeled **DOTA-EB-TATE** ^21,22^ justified first-in-human studies with the ^177^Lu-labeled analogue.^11–12^ To be consistent regarding the utilized radionuclide, we synthesized **[** ^**64**^ **Cu]­Cu-DOTA-EB-TATE**, which has not been investigated so far.

All ^64^Cu-labeled conjugates were subjected to a detailed *in vitro* and *in vivo* radiopharmacological characterization including the determination of the albumin- and SST_2_-binding affinities. For SST_2_ binding studies, a mouse pheochromocytoma cell line (MPC) with a high endogenous abundance of SST_2_ was used. In vivo distribution was investigated in a corresponding subcutaneous MPC allograft model.^23–25^

By reviewing the PET imaging results for the series of **[** ^**64**^ **Cu]­Cu-NODAGA-cLAB-TATEs** and of **[** ^**64**^ **Cu]­Cu-NODAGA-cLAB4-** *scr* **TATE**, we hypothesized that the increased integral tumor uptake for these heterobivalent radioligands might originate from the additional SST_2_-binding of the albumin-bound radioligand. To experimentally test this hypothesis, we analyzed saturation binding to SST_2_ on intact MPC cells for **[** ^**64**^ **Cu]­Cu-NODAGA-cLAB2-TATE** (as a model heterobivalent radioligand) in the presence of different albumin concentrations. Furthermore, we propose a dedicated three-compartment model for the quantitative description of the principal effects of albumin binding of radioligands in the blood circulation on the time course of excretion and tumor uptake when considering different tissue uptake rates of the free and albumin-bound radioligand, respectively. Analysis of this model and its solution supports our hypothesis and provides a rationale for the commonly observed increase in integral tumor uptake for heterobivalent radioligands with an albumin-binding moiety.

## Results and Discussion

### Synthesis and ^64^Cu labeling

The synthesis of **NODAGA-cLAB­(5–6)-TATEs** and **NODAGA-cLAB4-** *scr* **TATE** was performed according to our previously established synthesis strategy, which consists of the solid-phase synthesis of the precursor molecules **NODAGA-Pra-PEG2-TATE** and **NODAGA-Pra-PEG2-** *scr* **TATE** using standard conditions for Fmoc removal (20% piperidine/DMF) and amino acid coupling (HATU/DIPEA). This was followed by on-resin CuAAC of the respective cLABs (0.5 eq. CuSO_4_/THPTA and 5 eq. sodium ascorbate, ambient temperature). Subsequent acid-mediated cleavage from the resin and concomitant removal of the protecting groups provided the linear peptides, which were cyclized by stirring in an aqueous milieu in the presence of 10% DMSO at pH 7.0–8.0 for 24–48 h.^19^ To avoid the rather time-consuming cyclization in solution, we envisaged the on-resin cyclization by I_2_/DMF at the stage of the linear sequence (before PEG2 coupling).^26,27^ However, this led to incomplete conversions for the subsequent CuAAC and required the use of an excess of CuSO_4_ and THPTA (2 eq. each) compared to the amount of azide/alkyne and elevated temperatures (60 °C) for a satisfying reaction yield. In this context, it was previously shown that octreotide and SST14 are able to bind copper ions at two different binding sites, which involve either the disulfide bond or two aromatic residue (Phe3-d-Trp4 in octreotide and Phe6-Phe7 in SST14).^28,29^ Consequently, we hypothesize that the presence of the disulfide bridge might lower the amount of copper ions available for the CuAAC and thus an excess of copper becomes necessary for an efficient reaction. Of course, the on-resin cyclization could also be performed after the on-resin CuAAC, which was, however, not tested.

All peptides were obtained in sufficient overall yields (6–14%) and high chemical purities (≥95%, Table 1). ^64^Cu-labeling of the peptides was performed as described previously using in-house-produced [^64^Cu]­CuCl_2_ in ammonium acetate buffer at a pH value of around 5.5. Radiochemical yields for the peptides presented herein were usually greater than 98% at apparent molar activities between 25 and 40 GBq/μmol (Supporting Information).

**Table 1:** Analytical Data of the TATE Derivatives

Name	Chemical formula	*m*/*z* calculated for [M+2H]^2+^	*m*/*z* found for [M+2H]^2+^ ^a^	Purity (%)^b^
**NODAGA-cLAB5-TATE**	C_98_H_130_FN_21_O_26_S_2_	1051.4550	1051.4560	≥95
**NODAGA-cLAB6-TATE**	C_99_H_133_N_21_O_26_S_2_	1049.4676	1049.4684	≥96
**NODAGA-cLAB4-** *scr* **TATE**	C_99_H_133_N_21_O_26_S_2_	1049.4676	1049.4678	≥96

a High-resolution mass spectra using electrospray ionization were recorded.

b Purity was determined by analytical RP-HPLC and is given for 230 nm.

### Radiopharmacological Characterization *In Vitro*

#### HSA Binding and log D_7.4_ Values

The binding affinity of the ^64^Cu-labeled peptides to human serum albumin (HSA) was assessed with a radiometric ultrafiltration assay as previously described by us (Table 2 and Figure S1).^19^ As expected due to the use of N_3_Bz-l-Lys­(FPB)-NH_2_ as **cLAB5**, **[** ^**64**^ **Cu]­Cu-NODAGA-cLAB5-TATE** exhibits a really low binding affinity to albumin with a *K*_d_ value of 440 μM, which is close to the estimated value of 572 μM (Table S1) based on our previously explored structure–activity relationships for the isolated **cLABs**.^19^ The albumin-binding affinity of **[** ^**64**^ **Cu]­Cu-NODAGA-cLAB6-TATE**, for which N_3_Bz-d-Lys­(MPB)-NH_2_ was used as **cLAB6**, was determined to be 88 μM. This experimental result is again close to the predicted value of 132 μM (Table S1). In accordance with the albumin-binding affinity of **[** ^**64**^ **Cu]­Cu-NODAGA-cLAB4-TATE** (*K*_d_ = 50 μM), a *K*_d_ value of 42 μM was obtained for the peptide with the scrambled TATE sequence (**[** ^**64**^ **Cu]­Cu-NODAGA-cLAB4-** *scr* **TATE**). Overall, these data further support our previous finding that by using the **cLABs,** target molecules with predictable albumin-binding affinity can be designed and synthesized.

**Table 2:** Summary of *In Vitro* Data for the Different ^64^Cu-labeled TATE Derivatives

					*t*_**1/2**_ **(plasma, h)** ^a^	
Compound^b^	log D_7.4_ ^c^	*K*_d_ [HSA] (μM)^d^	*K*_d_ [SST_2_] (nM)^e^	*B*_max_ (fmol/mg of protein)^e^	Mouse	Human
**[** ^**64**^ **Cu]Cu-NODAGA-cLAB5-TATE**	–2.27 (0.13)	440 (190)	1.53 (0.40)	355 (53)	≈13	>24
**[** ^**64**^ **Cu]Cu-NODAGA-cLAB6-TATE**	–2.03 (0.13)	88 (14)	1.81 (0.09)	438 (16)	>24	>24
**[** ^**64**^ **Cu]Cu-NODAGA-cLAB4-** *scr* **TATE**	–2.17 (0.34)	42 (4)	n.b.	n.b.	>24	>24
**[** ^**64**^ **Cu]Cu-DOTA EB-TATE**	–2.26 (0.08)	1.2 (0.5)	19.5 (3.41)	247 (81)	n.d.	n.d.

a Plasma stability data are obtained in a single experiment. n.d. denotes not determined.

b After ^64^Cu-labeling, the nonmetalated ligand was not separated or saturated with ^nat^Cu^2+^.

c Data shown are mean values (±SD) of 3–9 separate processes of shaking out.

d Data shown are mean values (±SD) of 2–3 experiments (each experiment was performed in single execution) with radioligand concentrations of 2 or 20 μM.

e Data shown are mean values (±SEM) of 2–4 experiments, which were performed in triplicate. n.b. denotes no binding detected.

**[** ^**64**^ **Cu]­Cu-DOTA-EB-TATE** was also characterized for its binding affinity to HSA, and a *K*_d_ value of 1.2 μM has been determined. This result is in line with the binding affinity previously determined for nonlabeled **DOTA-EB-TATE** toward biotinylated BSA by biolayer interferometry (*K*_d_ = 4.8 μM).^21^

The log D_7.4_ values of the ^64^Cu-labeled peptides were determined with the octanol partitioning method. All peptides exhibit distribution coefficients in a narrow range between −2.03 and −2.27, which is in line with the data for the previously characterized ^64^Cu-labeled **NODAGA-cLAB-TATEs**. This demonstrates again that the introduction of the largely apolar albumin-binding moiety into **NODAGA-TATE** increases the lipophilicity by approximately one log unit (log D_7.4_ of −3.43 for **[** ^**64**^ **Cu]­Cu-NODAGA-TATE**).^19^

#### Plasma Stability

Previously, we discovered that the ^64^Cu-labeled **NODAGA-cLAB-TATEs** with a primary amide functionality within the albumin-binding moiety are subject to a metabolic transformation in mouse plasma. In fact, the primary amide is slowly hydrolyzed to the carboxylic acid, which turned out to be catalyzed by plasma carboxylesterases as proven by blocking this transformation in the presence of *bis*-para-nitrophenylphosphate.^19^ This metabolic transformation does not occur in human plasma *in vitro*, which originates from the fact, that the level of plasma carboxylesterases in human plasma is negligible.^30^ As this transformation entails also an increase in the binding affinity to albumin, there might be effects on the biodistribution in mice, in particular the blood circulation time. To investigate this, **NODAGA-cLAB6-TATE** was designed, with **cLAB6** (N_3_Bz-d-Lys­(MPB)-NH_2_) representing the d-configured pendant of **cLAB4** (N_3_Bz-l-Lys­(MPB)-NH_2_). In contrast to **[** ^**64**^ **Cu]­Cu-NODAGA-cLAB4-TATE**, **[** ^**64**^ **Cu]­Cu-NODAGA-cLAB6-TATE** is not hydrolyzed in mouse plasma (Figure 2) indicating the stereospecificity of plasma carboxylesterases for l-configured lysine carboxamides within these peptides. As expected, **[** ^**64**^ **Cu]­Cu-NODAGA-cLAB5-TATE** and **[** ^**64**^ **Cu]­Cu-NODAGA-cLAB4-** *scr* **TATE** (both bearing l-configured lysine carboxamides) are hydrolyzed in mouse plasma (but not in human plasma, Figure S2), albeit the rate of metabolic transformation is significantly lower for the latter (Figure 2). However, the apparent half-lives for both compounds (≈13 and >24 h) are longer compared to the half-lives previously determined for **[** ^**64**^ **Cu]­Cu-NODAGA-cLAB3-TATE** and **[** ^**64**^ **Cu]­Cu-NODAGA-cLAB4-TATE** (≈6 and ≈3 h, respectively).^19^ For better comparability, the stability of **[** ^**64**^ **Cu]­Cu-NODAGA-cLAB4-TATE** was again examined with the same mouse plasma batch used for **[** ^**64**^ **Cu]­Cu-NODAGA-cLAB5-TATE** and **[** ^**64**^ **Cu]­Cu-NODAGA-cLAB4-** *scr* **TATE**. The results revealed a similar half-life for **[** ^**64**^ **Cu]­Cu-NODAGA-cLAB4-TATE** of ≈12 h. Therefore, the discrepancy with our previously reported stability data in mouse plasma might result from differences in the activity of plasma carboxylesterases in the obtained mouse plasma. However, the rate of amide hydrolysis is indeed lower for **[** ^**64**^ **Cu]­Cu-NODAGA-cLAB4-** *scr* **TATE**, independent of the mouse plasma batch, which might indicate that the actual amino acid sequence in these peptides affects the recognition by the plasma carboxylesterases.

**Figure 2:**
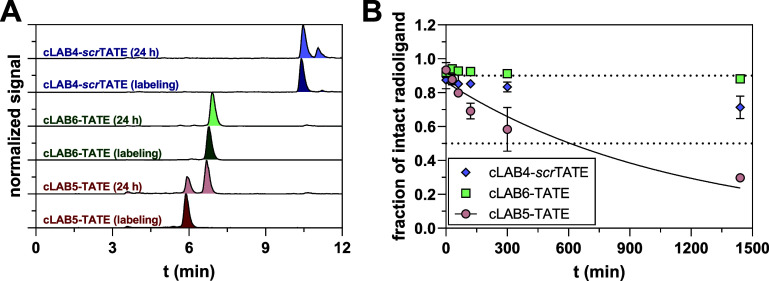
Stability of the ^64^Cu-labeled TATE derivatives in mouse plasma. (A) Radio-HPLC chromatograms of **[^64^Cu]­Cu-NODAGA-cLAB5-TATE** (red), **[^64^Cu]­Cu-NODAGA-cLAB6-TATE** (green), and **[^64^Cu]­Cu-NODAGA-cLAB-** *scr* **TATE** (blue) after ^64^Cu-labeling and incubation in mouse plasma for 24 h show the transformation of **[^64^Cu]­Cu-NODAGA-cLAB5-TATE** and **[^64^Cu]­Cu-NODAGA-cLAB4-** *scr* **TATE** into a metabolite of higher retention time (see the Discussion for explanation). (B) Plot of residual intact radioligands upon incubation with mouse plasma including nonlinear regression (black line) according to one-phase decay for **[^64^Cu]­Cu-NODAGA-cLAB5-TATE** (red circles). The dotted lines represent fractions of 0.5 and 0.9. Data shown are mean values (±SD) from 1 to 3 experiments (each experiment was performed in single execution). Plasma incubations were performed at 37 °C for 24 h, and aliquots were withdrawn at distinct time points (5/30 min and 2/4/24 h) for examining the metabolization of the radioligand.

#### SST_2_ Binding

Binding affinity to SST_2_ for the novel compounds was determined by saturation binding analysis using intact MPC cells (Table 2, saturation binding and standard curves are shown in Figures S3 and S4), which exhibit a high protein level of SST_2_.^23,24^ **[** ^**64**^ **Cu]­Cu-NODAGA-cLAB­(5/6)-TATEs** showed comparable binding affinities (1.53 and 1.81 nM, respectively) to previously characterized **[** ^**64**^ **Cu]­Cu-NODAGA-cLAB­(1/4)-TATE** (1.73 and 2.77 nM, respectively) and **[** ^**64**^ **Cu]­Cu-DOTA-TATE** (1.19 nM) and **[** ^**64**^ **Cu]­Cu-NODAGA-TATE** (1.43 nM) toward intact MPC cells.^31^ A diminished binding affinity was obtained for **[** ^**64**^ **Cu]­Cu-DOTA-EB-TATE** with a value of 19.5 nM. In line with our data, nonlabeled **DOTA-EB-TATE** was also less potent than **DOTA-TATE** in previous competition binding experiments using **[** ^**86**^ **Y]­Y-DOTA-EB-TATE** as a radioligand and intact AR42J cells (IC_50_ values of 9.16 and 3.46 nM, respectively) or SST_2_-transfected HTC116 cells (IC_50_ values of 16.50 and 10.21 nM, respectively) cells.^21^ In contrast, no binding was detected for **[** ^**64**^ **Cu]­Cu-NODAGA-cLAB4-** *scr* **TATE** (up to 40 nM), which proves that the scrambled sequence of TATE indeed abolished binding to SST_2_ as intended.

Furthermore, the receptor-mediated endocytosis (referred to as internalization) of the novel compounds was assessed. The values for the internalized fraction (or “acid wash” resistant fraction) were >50% (Figure S5), which might indicate a similar pharmacological behavior of these ligands upon binding to SST_2_ as the parent ligand TATE. **[** ^**64**^ **Cu]­Cu-NODAGA-cLAB4-** *scr* **TATE** showed no binding at all under the applied assay conditions.

#### PET Imaging

The biodistribution of **[** ^**64**^ **Cu]­Cu-NODAGA-cLAB­(5/6)-TATE**, **[** ^**64**^ **Cu]­Cu-NODAGA-cLAB4-** *scr* **TATE**, and **[** ^**64**^ **Cu]­Cu-DOTA-EB-TATE** was assessed in a subcutaneous MPC tumor allograft model via small-animal PET imaging (Figure 3A–D). The time-activity curves (TACs) for tumor, kidneys, and heart are shown in Figure 4A–D (for TACs of muscle and liver, see Figure S6) and the calculated areas under curves (AUC_0–48h_) and tumor-to-tissue ratios are graphically summarized in Figure 4E,F, respectively.

**Figure 3:**
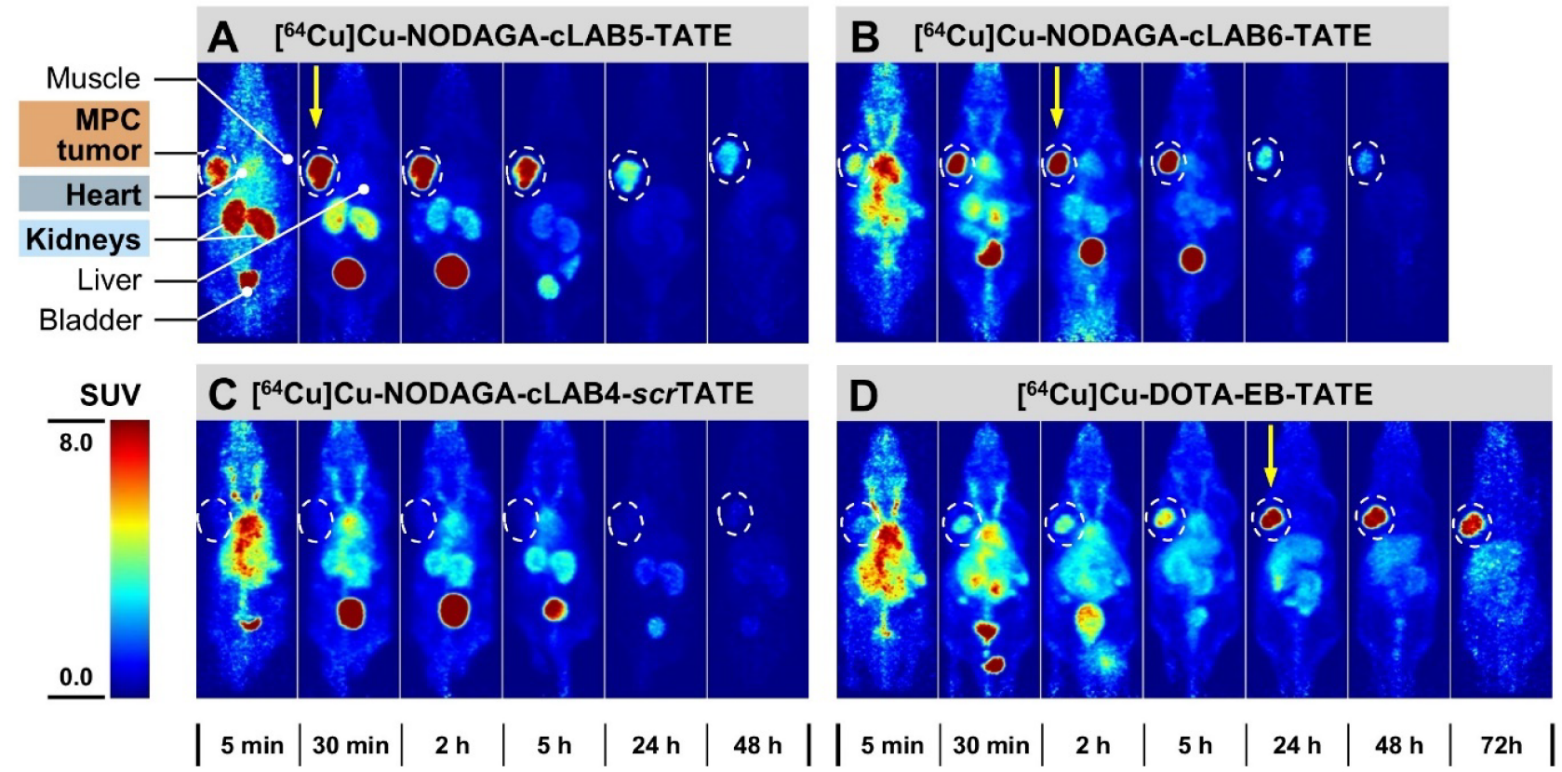
PET images for the ^64^Cu-labeled TATE derivative PET images (A–D) at selected time points after intravenous injection of the different ^64^Cu-labeled compounds (7–10 MBq/animal) in MPC tumor-bearing mice. Images are presented as maximum intensity projections and shown at common scale. Anatomical positions of the tumor, heart, kidney, liver, and muscle used for SUV quantification are exemplarily shown for [^64^Cu]­Cu-NODAGA-cLAB5-TATE (A). Dashed circles indicate tumor positions. Vertical arrows indicate time points with the highest tumor uptake. Indicated time points correspond to the following time frames: 5 min (4–6 min), 30 min (25–40 min), 2 h (105–120 min), 5 h (4.5–5.5 h), 24 h (23–25 h), and 48 h (46–50 h).

**Figure 4:**
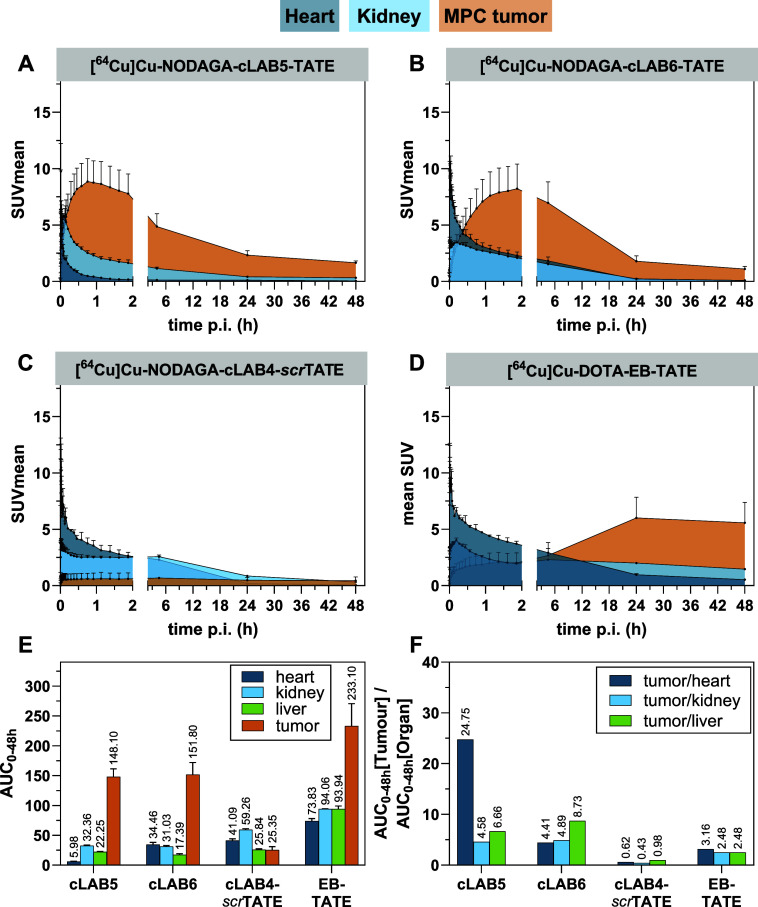
PET imaging data for the ^64^Cu-labeled TATE derivatives. (A–D) Time-activity curves (SUV, decay-corrected, as a function of time up to 48 h) obtained by PET acquisition are depicted (0–2 h in dynamic mode and time points 5, 24, and 48 h *p.i.*; the *x*-axes are segmented accordingly). Data shown are mean values (±SD) of a group of MPC tumor-bearing NMRI-nu/nu mice (*n* = 2, but for cLAB6-TATE *n* = 4). The area under the respective curves were filled with color for a better visualization. Same color coding as in ref 19. (E) Calculated AUC_0–48h_ values. Data shown are mean values (±SD). (F) Tumor/organ ratios based in the AUC_0–48h_ values. For a better overview in panels E and F, the compound names were abbreviated.

#### [^64^Cu]­Cu-NODAGA-cLAB5-TATE

**[** ^**64**^ **Cu]­Cu-NODAGA-cLAB5-TATE** showed a fast tumor uptake with a maximum SUVmean of 8.9 being reached at 45 min *p.i*., which remained largely constant up to 2 h *p.i.* (SUVmean of 7.9) before declining (SUVmean values of 4.9 and 2.3 after 4 and 24 h *p.i.*, respectively). The tumor TAC together with the TACs for the normal organs resemble the data obtained for **[** ^**64**^ **Cu]­Cu-NODAGA-TATE** resulting in comparable AUC_0–48h_ and tumor-to-tissue ratios (Figure S7). In this context, the low retention in blood (as assessed by the TAC for the heart) is in accordance with the negligible binding affinity of **[** ^**64**^ **Cu]­Cu-NODAGA-cLAB5-TATE** to albumin determined by the ultrafiltration assay (*K*_d_ = 440 μM). However, it is striking that the integral tumor uptake of **[** ^**64**^ **Cu]­Cu-NODAGA-cLAB5-TATE** is significantly higher than that observed for its alkyne precursor **[** ^**64**^ **Cu]­Cu-NODAGA-Pra-PEG2-TATE** (Figure S7, structure in Figure 1). Previously, we discussed that the clearance of **[** ^**64**^ **Cu]­Cu-NODAGA-Pra-PEG2-TATE** from the organism is faster compared to **[** ^**64**^ **Cu]­Cu-NODAGA-TATE**, which results in a lower integral kidney and tumor uptake, but almost identical tumor-to-organ ratios.^19,27^ Consequently, the faster clearance conferred by the hydrophilic Pra-PEG2 unit seems to be compensated upon combination with **cLAB5**, which is most likely independent of the albumin-binding capability.

#### [^64^Cu]­Cu-NODAGA-cLAB6-TATE

Compared to **[** ^**64**^ **Cu]­Cu-NODAGA-cLAB5-TATE**, the blood circulation time of **[** ^**64**^ **Cu]­Cu-NODAGA-cLAB6-TATE** was significantly prolonged, which is accompanied by a protracted tumor uptake (SUVmean of 8.2 being reached after 2 h *p.i*., Figure S8). While the albumin-binding affinity of **[** ^**64**^ **Cu]­Cu-NODAGA-cLAB6-TATE** was slightly lower than that of **[** ^**64**^ **Cu]­Cu-NODAGA-cLAB4-TATE** (88 vs 50 μM), there was no clear difference in the heart TAC (Figure S8). Consequently, this indicates that the hydrolysis of the primary amide functionality in **[** ^**64**^ **Cu]­Cu-NODAGA-cLAB4-TATE** by plasma carboxylesterases does not substantially affect the biodistribution. In accord with the similarity of the heart TACs, the tumor TACs were also similar up to 2 h *p.i.* for both compounds. However, later, the tumor accumulation of **[** ^**64**^ **Cu]­Cu-NODAGA-cLAB6-TATE** already declined, while the tumor uptake of **[** ^**64**^ **Cu]­Cu-NODAGA-cLAB4-TATE** further increased up to 5 h *p.i.*, reaching a maximum SUVmean of 10.2. Furthermore, the SUVmean values at 24 and 48 h *p.i.* were significantly lower for **[** ^**64**^ **Cu]­Cu-NODAGA-cLAB6-TATE** (1.8 and 1.1) compared to **[** ^**64**^ **Cu]­Cu-NODAGA-cLAB4-TATE** (5.5 and 2.4). Consequently, for **[** ^**64**^ **Cu]­Cu-NODAGA-cLAB4-TATE**, a higher AUC_0–48h_[tumor] was reached compared to **[** ^**64**^ **Cu]­Cu-NODAGA-cLAB6-TATE** (289 and 152 SUVmean*h, respectively, Figure S9). This in turn indicates that the configuration within the albumin-binding moiety affects the tumor residence time with a d-configured lysine leading to a shorter residence time than a l-configured lysine. This is further supported by data for the cellular release of both compounds after initial binding to intact MPC cells (Figure S10). In fact, 24 h after radioligand removal, 24% of initially cell-bound **[** ^**64**^ **Cu]­Cu-NODAGA-cLAB4-TATE** were still bound,^20^ but only 11% of **[** ^**64**^ **Cu]­Cu-NODAGA-cLAB6-TATE**. In contrast, the cellular release of **[** ^**64**^ **Cu]­Cu-NODAGA-TATE** was comparable to that of **[** ^**64**^ **Cu]­Cu-NODAGA-cLAB4-TATE** indicating that structural modifications at the N-terminus of TATE exert not generally unfavorable effects on the cellular retention.^20^ The particular reason for the dependence of the cellular retention on the configuration of the albumin-binding moiety cannot be given at this stage, but it might originate from a different receptor trafficking behavior and/or receptor dissociation rate constants due to a different spatial orientation of the albumin-binding moiety within the SST_2_ binding pocket.

With **[** ^**64**^ **Cu]­Cu-NODAGA-cLAB5/6-TATE**, we formally conclude the series of **[** ^**64**^ **Cu]­Cu-NODAGA-cLAB-TATEs** (Figure 1). Among these six heterobivalent (SST_2_/albumin) radioligands, **[** ^**64**^ **Cu]­Cu-NODAGA-cLAB4-TATE** with an albumin-binding affinity of 50 μM is clearly the radioligand with the best *in vivo* performance in terms of integral tumor uptake over 48 h and tumor-to-organ ratios. In tumor tissue, this radioligand exhibits the highest SUVmean peak, the highest AUC_0–48h_ and also one of the highest SUVmean at 48 h *p.i.* (Figure S11). AUC_0–48h_[Heart] is increased by a factor of 7.6 compared to **[** ^**64**^ **Cu]­Cu-NODAGA-TATE** with the increase being caused by a distinct broadening of the time-activity curve. In contrast, its integral kidney uptake is comparable to that of **[** ^**64**^ **Cu]­Cu-NODAGA-TATE**, albeit a much lower maximum SUVmean is observed. The favorable pharmacokinetics of **[** ^**64**^ **Cu]­Cu-NODAGA-cLAB4-TATE** prompted us recently to characterize its ^67^Cu-labeled analog in preclinical therapy studies in comparison to **[** ^**67**^ **Cu]­Cu-NODAGA-TATE** and **[** ^**177**^ **Lu]­Lu-DOTA-TATE**.^20^ It is worth noting that also upon ^67^Cu-labeling, the integral tumor uptake of **[** ^**67**^ **Cu]­Cu-NODAGA-cLAB4-TATE** was improved compared to **[** ^**67**^ **Cu]­Cu-NODAGA-TATE** and even an equivalent treatment efficacy to **[** ^**177**^ **Lu]­Lu-DOTA-TATE** was achieved. The latter finding appears actually surprising as **[** ^**177**^ **Lu]­Lu-DOTA-TATE** exhibits also no dedicated albumin-binding moiety and we initially expected that **[** ^**67**^ **Cu]­Cu-NODAGA-cLAB4-TATE** will outperform **[** ^**177**^ **Lu]­Lu-DOTA-TATE**. However, for some reasons, the metal–chelator combination [^177^Lu]­Lu-DOTA leads to a favorable tumor cell retention, which is much longer compared to the ^67^Cu-labeled **NODAGA-TATE** and **NODAGA-cLAB-TATEs** and mainly explains its superior *in vivo* performance.

#### [^64^Cu]­Cu-NODAGA-cLAB4-scrTATE

To gain insight into the tumor uptake of the albumin-bound radioligand and/or off-target binding by the albumin-binding moiety in the absence of SST_2_-binding, **[** ^**64**^ **Cu]­Cu-NODAGA-cLAB4-** *scr* **TATE** was designed. Owing to the same albumin-binding moiety (**cLAB4**) and comparable physiochemical properties—as only the sequence and not the identity of the amino acids was changed—the overall biodistribution of **[** ^**64**^ **Cu]­Cu-NODAGA-cLAB4-** *scr* **TATE** resembles that of **[** ^**64**^ **Cu]­Cu-NODAGA-cLAB4-TATE**. However, the integral tumor uptake is reduced to less than 10% of the tumor uptake observed for **[** ^**64**^ **Cu]­Cu-NODAGA-cLAB4-TATE** (AUC_0–48h_ of 25 and 289 SUVmean*h, respectively). Furthermore, the TAC for the tumor uptake of **[** ^**64**^ **Cu]­Cu-NODAGA-cLAB4-** *scr* **TATE** resembled a constant background level of activity uptake with comparable SUVmean values as observed for the muscle tissue. However, the uptake in the tumor tissue is significantly higher compared to the muscle at later time points *p.i.* (e.g., 0.49 vs 0.04 for tumor and muscle at 24 h *p.i.*, respectively), which overall led to a higher integral uptake (AUC_0–48h_ of 25 and 5.5 SUVmean*h for tumor and muscle, respectively). This indicates that a certain tumor retention occurs by **[** ^**64**^ **Cu]­Cu-NODAGA-cLAB4-** *scr* **TATE** but this does not explain the increased integral tumor uptake observed for **[** ^**64**^ **Cu]­Cu-NODAGA-cLAB4-TATE** compared to **[** ^**64**^ **Cu]­Cu-NODAGA-TATE** or **[** ^**64**^ **Cu]­Cu-NODAGA-cLAB5-TATE**. Consequently, the existence of an SST_2_-dependent mechanism likely explains the increased tumor uptake.

The design of the control peptide **NODAGA-cLAB4-** *scr* **TATE** followed the approach previously reported by Chen et al.^32^ who synthesized and characterized a cyclic pentapeptide with a central RAD sequence instead of RGD (^**64**^ **Cu-NMEB-RAD**) as a control peptide (no cell binding *in vitro*) for their α_v_β_3_-targeting truncated Evans Blue/RGD conjugate (^**64**^ **Cu-NMEB-RGD**). In contrast to the lower integral tumor uptake of **[** ^**64**^ **Cu]­Cu-NODAGA-cLAB4-** *scr* **TATE** in comparison to **[** ^**64**^ **Cu]­Cu-NODAGA-cLAB4-TATE** (11-fold lower) but also **[** ^**64**^ **Cu]­Cu-NODAGA-TATE** (6-fold lower), the integral tumor uptake of the nontargeting ^**64**^ **Cu-NMEB-RAD** was only 3-fold lower compared to ^**64**^ **Cu-NMEB-RGD** but even 4-fold higher compared to ^**64**^ **Cu-NOTA-RGD** (radioligand without NMEB as albumin-binding moiety). The authors discuss that a pronounced EPR effect is responsible for the high uptake of ^**64**^ **Cu-NMEB-RAD**, but apparently another mechanism must exist that accounts for the improved performance of ^**64**^ **Cu-NMEB-RGD** compared to ^**64**^ **Cu-NOTA-RGD**.

As we have previously shown, there was a fairly linear relationship between binding affinity to HSA and the retention in blood (AUC_0–48 h_) for **[** ^**64**^ **Cu]­Cu-NODAGA-cLAB­(1–4)-TATE**.^19^ It is worth noting that the data for **[** ^**64**^ **Cu]­Cu-NODAGA-cLAB­(5/6)-TATE** and **[** ^**64**^ **Cu]­Cu-NODAGA-cLAB4-** *scr* **TATE** fit well into this linear relationship (Figure S12).

#### [^64^Cu]­Cu-DOTA-EB-TATE

Previously, Xiaoyuan Chen and colleagues developed **DOTA-EB-TATE**, which bears a truncated Evans blue (EB) moiety for reversible binding to albumin. ^86/90^Y- and ^177^Lu-labeled **DOTA-EB-TATE** were preclinically characterized and **[** ^**177**^ **Lu]­Lu-DOTA-EB-TATE** has also entered clinical therapy trials.,^10,12,13^ Recently, also **[** ^**67**^ **Cu]­Cu-DOTA-EB-TATE** has been investigated for its theranostic efficacy in preclinical studies.^33^ Based on our motivation to perform a side-by-side comparison to the series of **NODAGA-cLAB-TATEs**, we performed the ^64^Cu-labeling of **DOTA-EB-TATE** and characterized the radioligand using the subcutaneous MPC tumor allograft model.

Due to the high albumin-binding affinity of **[** ^**64**^ **Cu]­Cu-DOTA-EB-TATE** (*K*_d_ = 1.2 μM, Table 2), the blood retention is prolonged and the tumor uptake is protracted compared to radioligands such as **[** ^**64**^ **Cu]­Cu-NODAGA-cLAB5-TATE** or **[** ^**64**^ **Cu]­Cu-NODAGA-TATE**. In fact, the TACs for heart and tumor uptake resemble those for **[** ^**64**^ **Cu]­Cu-NODAGA-cLAB2-TATE**, which exhibits a similar binding affinity to albumin (*K*_d_ = 1.8 μM). However, at 24 and 48 h p.i., the SUVmean values for **[** ^**64**^ **Cu]­Cu-DOTA-EB-TATE** were significantly higher compared to **[** ^**64**^ **Cu]­Cu-NODAGA-cLAB2-TATE** (6.0 vs 3.1 at 24 h and 5.6 and 2.4 at 48 h *p.i.*, Figure S13). Even at 72 h *p.i.*, the tumor uptake of **[** ^**64**^ **Cu]­Cu-DOTA-EB-TATE** remained largely unchanged (SUVmean of 5.6, Figure S12). This indicates a better retention of **[** ^**64**^ **Cu]­Cu-DOTA-EB-TATE** in the tumor tissue and resulted in an almost doubling of the AUC_0–48h_[Tumor] (233 vs 123 SUVmean*h) compared to **[** ^**64**^ **Cu]­Cu-NODAGA-cLAB2-TATE**. However, along with the doubled integral tumor uptake, the integral kidney and liver uptake also almost doubled compared to **[** ^**64**^ **Cu]­Cu-NODAGA-cLAB2-TATE**. While the latter can be explained by the lower kinetic inertness of the [^64^Cu]­Cu-DOTA complex compared to the [^64^Cu]­Cu-NODAGA complex in mice,^34^ the higher kidney uptake is known for EB-functionalized molecules and seems to be an inherent phenomenon. In this context, recent structural optimizations in EB-functionalized molecules by substituting the thiosuccinimide linkage with a PEG-linker further improved the tumor uptake but did not lower the kidney uptake.^35^ Owing to its higher integral tumor uptake over the first 48 h *p.i.* and simultaneously lower blood retention as well as kidney uptake (Figure S14), the *in vivo* performance of **[** ^**64**^ **Cu]­Cu-NODAGA-cLAB4-TATE** in the MPC tumor allograft model appears to be superior in comparison to **[** ^**64**^ **Cu]­Cu-DOTA-EB-TATE**.

### Assessing the Mechanisms for the Increased Tumor Uptake

#### SST_2_ Binding in the Presence of HSA

Reviewing the PET imaging data for the series of **[** ^**64**^ **Cu]­Cu-NODAGA-cLAB-TATEs** and **[** ^**64**^ **Cu]­Cu-NODAGA-cLAB4-** *scr* **TATE**, it becomes clear that the presence of a SST_2_-independent tumor uptake mechanism cannot sufficiently explain the increased integral tumor uptake compared to radioligands without a pronounced albumin-binding capability and even similar structural features (**[** ^**64**^ **Cu]­Cu-NODAGA-TATE** and **[** ^**64**^ **Cu]­Cu-NODAGA-cLAB5-TATE**). Consequently, we hypothesize that additional SST_2_-dependent binding occurs at the tumor sites. Upon injection of the **[** ^**64**^ **Cu]­Cu-NODAGA-cLAB-TATEs** (as well as other heterobivalent radioligands), the existence of two different molecular species in the blood circulation can be assumed that are in a dynamic equilibrium: the free and albumin-bound radioligand. A likely explanation for the increased tumor uptake might therefore be that the albumin-bound radioligand is also able to bind to SST_2_, which means a ternary complex is formed, at least initially. The potential target binding ability of the albumin-bound radioligand was previously mentioned by Eder et al.^36^ for a ^177^Lu-labeled bicyclic peptide (bearing a palmitoyl residue as albumin binder) targeting the matrix metalloproteinase MT1-MMP. For assessing the SST_2_-binding capability of the albumin-bound **NODAGA-cLAB-TATEs**, we envisaged a series of *in vitro* saturation binding analyses in the presence of varying concentrations of HSA. In case the albumin-bound radioligand is not SST_2_ reactive, the apparent binding affinity to SST_2_ (*K*_d_[SST_2_]) should proportionally decrease with increasing HSA concentrations. In contrast, in case of a possible binding of the albumin-bound radioligand to SST_2_, the binding affinity should not change (if free and albumin-bound radioligand are comparably affine for SST_2_) or should decrease to a threshold value (if the binding affinity of the albumin-bound radioligand is lower than that of the free radioligand). We decided to perform these experiments with the previously developed **[** ^**64**^ **Cu]­Cu-NODAGA-cLAB2-TATE** (Figure 1).^19^ This radioligand exhibits the best binding affinity to albumin (*K*_d_ = 1.8 μM) within the series of ^64^Cu-labeled **NODAGA-cLAB-TATEs**, which enables *in vitro* investigations up to really low f_u_ values at moderately high albumin concentrations (f_u_ = 0.02 at 100 μM albumin). In case of **[** ^**64**^ **Cu]­Cu-NODAGA-cLAB4-TATE** (*K*_d_[HSA] = 50 μM), we would have had to use nonphysiological high concentrations of albumin (≥500 μM) to obtain f_u_ values of at least <0.1. We assumed that such high albumin concentrations would generally interfere with cell binding of the radioligand apart from lowering the free fraction of the radioligand. Since within the series of **NODAGA-cLAB-TATEs** the structural changes are minimal and limited to the albumin-binding moiety (varying substituent at the phenyl ring and/or lysine carboxylic acid/carboxamide functionality, Figure 1), we hypothesized that the results obtained for **[** ^**64**^ **Cu]­Cu-NODAGA-cLAB2-TATE** are representative for the entire compound series. All members of the **NODAGA-cLAB-TATEs** are heterobivalent radioligands and they share the same binding site at albumin, which might justify to extrapolate the results for **[** ^**64**^ **Cu]­Cu-NODAGA-cLAB2-TATE** to the other analogs. In addition to **[** ^**64**^ **Cu]­Cu-NODAGA-cLAB2-TATE**, we included **[** ^**64**^ **Cu]­Cu-NODAGA-TATE** in these experiments, which served as reference ligand with no pronounced albumin-binding ability.

The saturation binding experiments were performed in the presence of 1, 5, 20, 40, and 100 μM HSA, which correspond to *f*_u_ values of 0.64, 0.26, 0.08, 0.04, and 0.02, according to the relation f_u_=*K*_d_/(Bmax+*K*_d_).^37^ Blakeley et al.^38^ developed a mathematical model that includes radioligand, protein (herein HSA), and target receptor (herein SST_2_) to derive the *K*_d_[HSA] value of the radioligand from saturation binding experiments to the target receptor in the presence of HSA. The authors specified an explicit mathematical solution for their proposed cubic equation and provided this solution also in a practical format for GraphPad Prism, which allows the analysis of experimental data but also simulations. For the binding of **[** ^**64**^ **Cu]­Cu-NODAGA-cLAB2-TATE** to SST_2_, we conducted ligand–receptor occupancy simulations with the previously determined *K*_d_[HSA] value of 1.8 μM,^19^ the herein determined *K*_d_[SST_2_] value of 3.4 nM using intact MPC cells and the HSA concentrations 1, 5, 20, 40, and 100 μM (Figures 5A and S15 and S16). As expected, a parallel rightward shift in the saturation binding curves was obtained in the presence of HSA. The respective apparent *K*_d_[SST_2_] values are listed in Table 3. Experimentally determined saturation binding curves of one experiment for **[** ^**64**^ **Cu]­Cu-NODAGA-cLAB2-TATE** and **[** ^**64**^ **Cu]­Cu-NODAGA-TATE** are shown in Figure 5B,C, respectively. Similar to the simulation, the experimentally recorded saturation binding curves for **[** ^**64**^ **Cu]­Cu-NODAGA-cLAB2-TATE** are significantly rightward-shifted, resulting in higher apparent *K*_d_[SST_2_] values in the presence of HSA compared to the conditions without HSA. However, the obtained *K*_d_[SST_2_] values were lower compared to the simulation data. This difference became more pronounced at higher HSA concentrations (*K*_d_[SST_2_] of 193 vs 58.9 nM at 100 μM HSA). For **[** ^**64**^ **Cu]­Cu-NODAGA-TATE**, albeit a slight rightward shift in the saturation binding curves in the presence of HSA was detectable, there seems to be no dependence on the applied HSA concentration.

**Figure 5:**
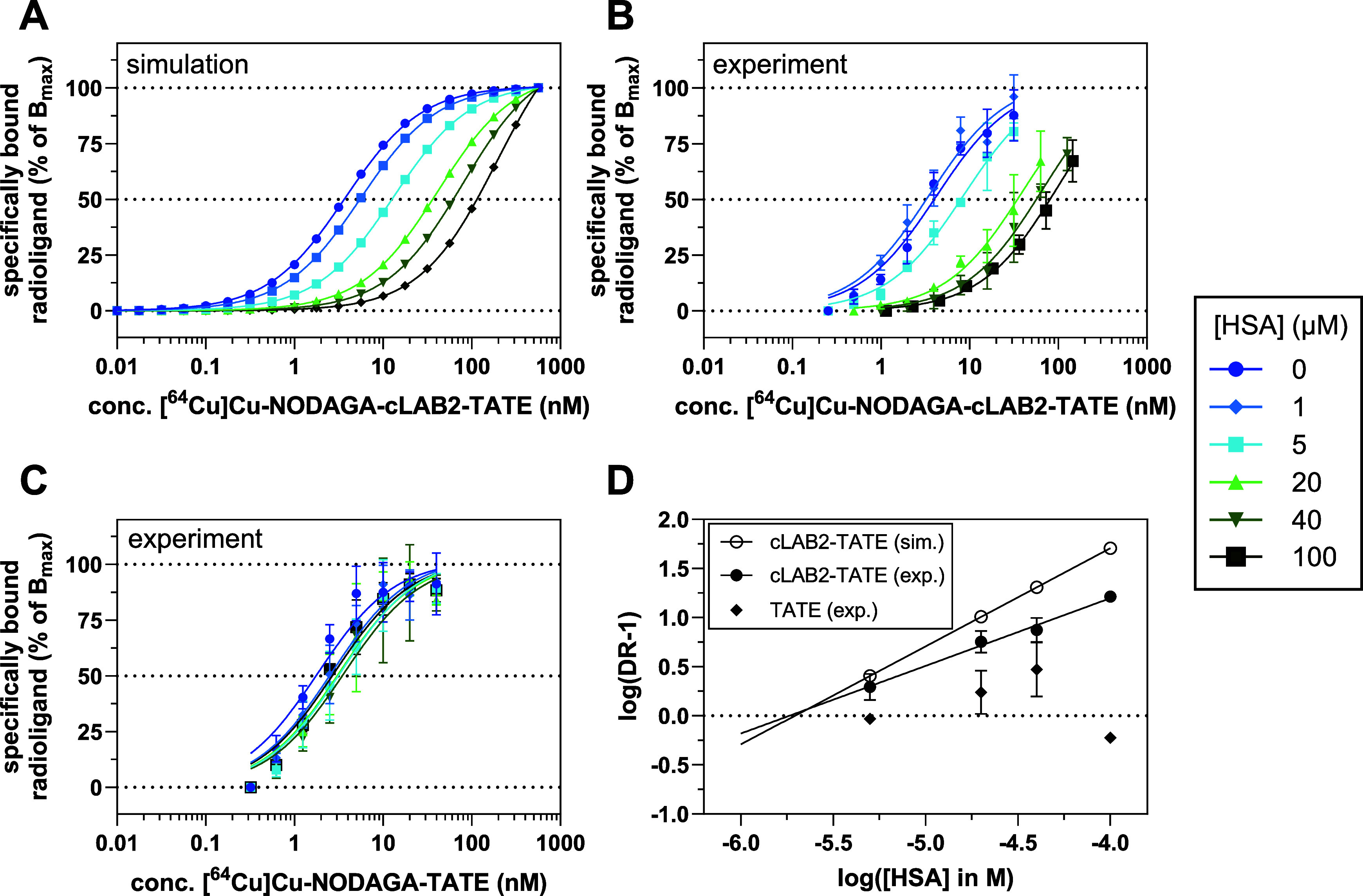
Schild analysis of SST_2_ binding in the presence of HAS. (A) Simulation of the effects of increasing concentration of HSA on the saturation binding of [^64^Cu]­Cu-NODAGA-cLAB2-TATE for the parameter configuration *K*_d_[SST_2_] = 3.4 nM, *K*_d_[HSA] = 1.8 μM, and [SST_2_]_T_ = 0.5 nM. Simulation was performed in GraphPad Prism (version 10.2.3) according to the solution in PRISM language provided by Blakeley et al.^38^ for their derived cubic equation. (B, C) Exemplary saturation binding curves of [^64^Cu]­Cu-NODAGA-cLAB2-TATE (B) and [^64^Cu]­Cu-NODAGA-TATE (C) to intact MPC cells in the presence of increasing concentrations of HSA. For each curve, data shown are mean values (±SD) of one experiment, which was performed in triplicate. (D) The *K*_d_ values (see Table 3, for *B*_max_ values, see Figure S17) obtained by the saturation binding analyses were used to calculate the log­(DR-1) values (see the Experimental Section for further details, data for 1 μM HSA were excluded as the value for log­(DR-1) based on the simulation data would be negative), which were plotted against log­([HSA]) (Schild plot). This plot was analyzed by linear regression. The simulation data provide a slope of unity, while for [^64^Cu]­Cu-NODAGA-cLAB2-TATE, a slope of 0.69 (CI of 95%: 0.48–0.90, *R* ^2^ = 0.990) was obtained. Based on the intercept at the *x*-axis, a *K*_d_[HSA] value of 1.8 μM was calculated for the experimental data of [^64^Cu]­Cu-NODAGA-cLAB2-TATE, which is well in accordance with the value determined with an ultrafiltration assay (1.8 μM).^19^

**Table 3:** Summary of *K*_d_[SST_2_] Values Using Intact MPC Cells in the Presence of Varying HSA Concentrations and Summary of the *K*_d_[HSA] Values Derived Thereof^a^

	*K*_d_[SST_2_] (nM)			*K*_d_[HSA] (μM)^**#**^	
[HSA] (μM)	cLAB2-TATE simulation	cLAB2-TATE experiment	TATE experiment	cLAB2-TATE	TATE
**0**	3.72	3.40 (0.08)	1.26 (1.09–1.45)	-	-
**1**	5.62	3.01 (0.45)	1.41 (0.40)	-	5.63 (4.30)
**5**	13.2	10.4 (2.1)	1.53 (0.91)	3.01 (1.05)	4.02
**20**	41.7	23.4 (5.0)	3.73 (1.16)	3.06 (0.87)	3.89 (2.66)
**40**	79.6	29.8 (7.3)	5.78 (2.54)	2.59 (1.14)	16.7 (9.4)
**100**	193	58.9 (1.0)	2.02 (0.06)	6.12 (1.02)	108 (7)

a Data shown are mean values (±SEM) of 2 experiments or mean values (CI, 68%) of 1 experiment with each experiment performed in triplicate. ^#^ *K*_d_[HSA] values were derived from the saturation binding experiments according to nonlinear regression using the model from Blakeley et al.^38^ with the following constrained parameters: *K*_D_[SST_2_] = 3.40 nM for cLAB2-TATE and 1.26 nM for TATE; [SST_2_]_T_ was calculated specifically for each experiment (usually <1 nM); [HSA]_T_ = 1, 5, 20, 40, and 100 μM.

Analyzing the experimental data by nonlinear regression according to the model of Blakeley et al.^38^ with fixed values for [HSA], [SST_2_], and *K*_d_[SST_2_], provided the *K*_d_[HSA] values (Table 3). A rather narrow range between 2.6 and 6.1 μM was obtained for **[** ^**64**^ **Cu]­Cu-NODAGA-cLAB2-TATE**, while these values were between 3.9 and 108 μM for **[** ^**64**^ **Cu]­Cu-NODAGA-TATE**. In addition to the aforementioned analyses, Blakeley et al.^38^ demonstrated that according to their mathematical model the binding affinity of ligands to plasma proteins can be determined by Schild analysis. For the present experiments, the Schild plot is shown in Figure 5D for both the experimental and simulation data. For **[** ^**64**^ **Cu]­Cu-NODAGA-cLAB2-TATE**, a *K*_d_[HSA] value of 1.8 μM was determined, while for **[** ^**64**^ **Cu]­Cu-NODAGA-TATE** the data points clearly deviate from linearity.

Our analysis so far seems to indicate that albumin-bound **[** ^**64**^ **Cu]­Cu-NODAGA-cLAB2-TATE** is not able to bind to SST_2_ as the *K*_d_ values increase with increasing HSA concentration and the *K*_d_[HSA] value derived by Schild analysis is in accordance with the binding affinity determined with the ultrafiltration assay. However, an important prerequisite by definition for accurately performing the Schild analysis is that the slope of the linear line should approach unity, which is clearly not the case for **[** ^**64**^ **Cu]­Cu-NODAGA-cLAB2-TATE** (slope of 0.69). This deviation is a consequence of the finding that the determined *K*_d_[SST_2_] values approach a threshold value with increasing HSA concentrations (≥60 nM, Figure S17). We conclude that actually albumin-bound **[** ^**64**^ **Cu]­Cu-NODAGA-cLAB2-TATE** is SST_2_-reactive resulting in the initial formation of a ternary complex (HSA-peptide-SST_2_), but the binding affinity of the albumin-bound radioligand is at least by a factor of 20 lower compared to the free radioligand, i.e., in the absence of HSA. In this context, Peng et al.^39^ previously developed albumin fusion proteins in which two molecules of either SST14 or SST28 are N-terminally attached to HSA. The respective fusion proteins (SST14)_2_-HSA and (SST28)_2_-HSA are still able to bind to all members of the SST receptor family as determined by competition binding experiments using [^125^I]­I-Tyr^1^-somatostatin as radioligand. Toward SST_2_, the IC_50_ values were 22.4 and 56.8 nM for (SST14)_2_-HSA and (SST28)_2_-HSA, respectively, while the IC_50_ value for SST14 was 2.77 nM. Thus, these results are concordant with our own. It is worth noting that the middle SST14 or SST28 molecule (directly attached to HSA) in these fusion proteins will certainly not be able to bind to SST receptors but rather act as a linker/spacer between the N-terminal peptides and HSA. Furthermore, Kazuta et al.^40^ recently reported on a PSMA-targeting radioligand covalently attached to HSA (**[** ^**111**^ **In]­In-PNT-DM-HSA**, attached to Cys34 of albumin via a thiosuccinimide group). Although no saturation binding analysis was performed, binding studies to LNCaP and PC-3 cells indicate that **[** ^**111**^ **In]­In-PNT-DM-HSA** is able to specifically bind to PSMA, albeit to a much lower extent compared to its maleimide precursor **[** ^**111**^ **In]­In-PNT-DM** or **[** ^**111**^ **In]­In-PSMA-617**. Accordingly, tumor uptake *in vivo* was lower compared to **[** ^**111**^ **In]­In-PNT-DM** at all investigated time points (4, 24, and 48 h *p.i.*). Consequently, the putative binding of albumin-bound **[** ^**64**^ **Cu]­Cu-NODAGA-cLAB2-TATE** to SST_2_ appears to be reasonable. As mentioned above, due to the high structural similarity (Figure 1), the binding ability to SST_2_ of albumin-bound **[** ^**64**^ **Cu]­Cu-NODAGA-cLAB2-TATE** might be representative for the other members of the series of **[** ^**64**^ **Cu]­Cu-NODAGA-cLAB-TATEs**.

#### A Compartment Model for the Description of the Influence of Albumin Binding on Tumor Uptake

The apparent findings that both free and albumin-bound radioligand are able to bind to SST_2_ provide a tentative qualitative explanation for the increased integral tumor uptake for **[** ^**64**^ **Cu]­Cu-NODAGA-cLAB4-TATE** in comparison to **[** ^**64**^ **Cu]­Cu-NODAGA-TATE** and **[** ^**64**^ **Cu]­Cu-NODAGA-cLAB5-TATE**. To further support this hypothesis, we sought to analyze the principal effects of albumin binding on excretion and tumor uptake of the radioligand from the compartment modeling perspective.

We propose the model structure shown in Figure 6A which corresponds to the system of coupled linear differential equations shown in Figure 6B. In contrast to the usual situation in PET compartment modeling where the arterial input function (AIF) is assumed to be known/measured and only the different tissue compartments in which the radioligand can reside are considered “true” compartments whose time course needs to be modeled, we treat the whole central blood pool as a proper compartment which remains “well stirred” at all times and presume that no relevant differences exist between the local arterial radioligand concentration in the vicinity of the tumor and the central blood pool. For the sake of simplicity, we also treat the input/injection at *t* = 0 as instantaneous, i.e., we are considering the (unit) impulse response of the compartment model. Given the relevant time scale/slow kinetics, this is a very good approximation of the actual experimental situation, but it would of course be straightforward, if need be, to convolve the derived impulse response with any given shape of the actual finite-duration injection. For completeness, we also note that the set of differential equations in Figure 6B requires referring all concentrations to a common volume (which might naturally be taken to coincide with the volume of the central blood pool) or requires considering the quantities C_l_, C_u_, C_b_ to represent (radioligand) amounts rather than concentrations. Therefore, C_u_ + C_b_ is proportional but not identical to the local radioligand concentration in the tumor as measured in a PET experiment.

**Figure 6:**
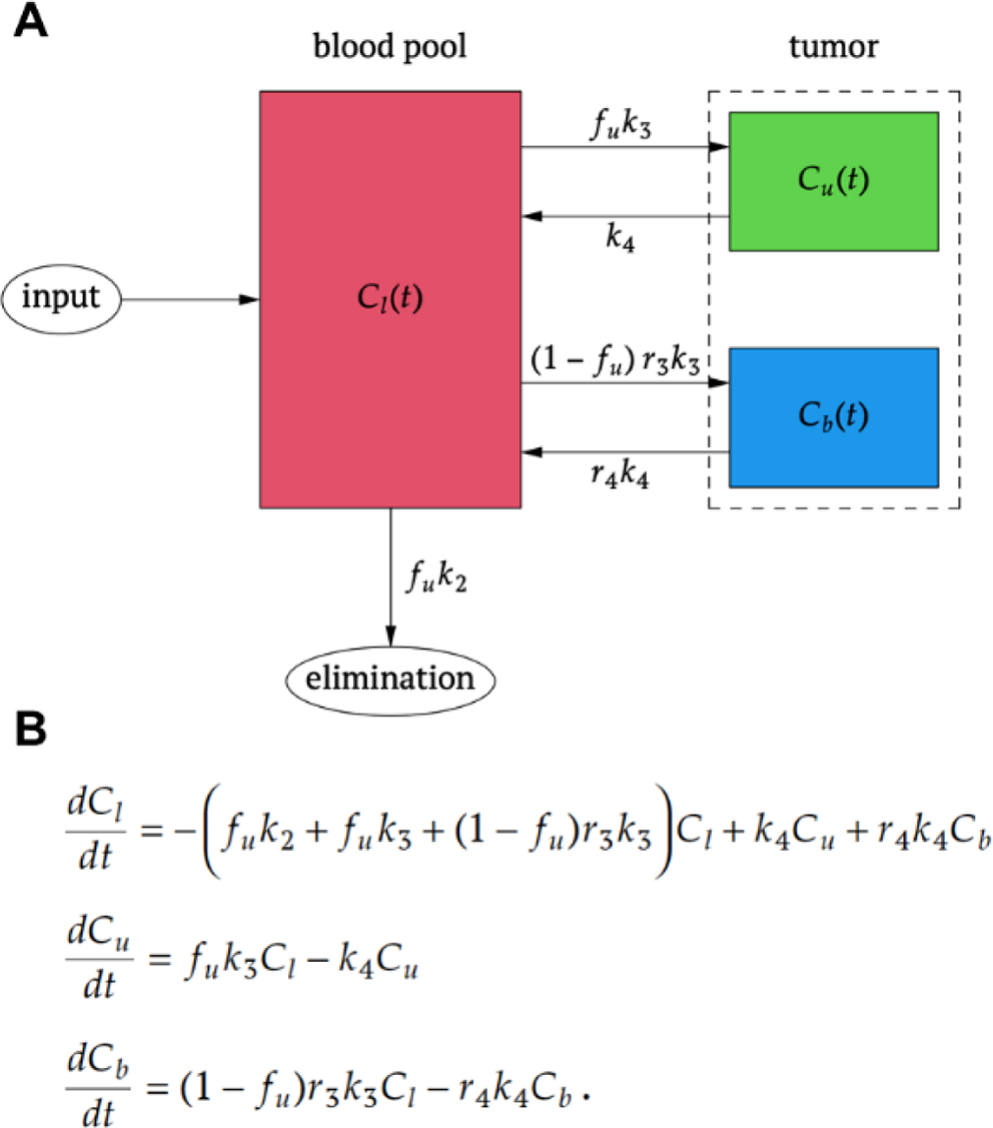
Three-compartment model for the description of ligand kinetics in the presence of albumin binding. (A) Model configuration: total radioligand in blood, *C*_l_; free (unbound) radioligand in tumor, *C*_u_; albumin-bound radioligand in tumor, *C*_b_; fraction of free radioligand, *f*_u_; renal excretion rate constant of free ligand, *k*_2_; tissue uptake rate constant of free ligand, *k*_3_; tissue clearance rate constant of free ligand, *k*_4_; and transport constant ratios r_3_ and r_4_ for describing the difference in tissue uptake and clearance rate, respectively, between free and albumin-bound radioligand. (B) System of coupled linear differential equations corresponding to the model in panel A.

Formally, the considered model is a coupled three-compartment model with six free parameters: fraction of free ligand (not bound to albumin) in blood, *f*_u_, renal excretion rate constant of free ligand only (absence of any albumin-binding), *k*_2_, tissue uptake and washout rate constants of free ligand only, *k*_3_ and *k*_4_, (which might tentatively be identified with the receptor association and dissociation rate constants) and transport constant ratios *r*_3_ and *r*_4_ which describe the difference in tissue uptake and tissue clearance between free and albumin-bound ligand, i.e., *r*_3_ × *k*_3_ and *r*_4_ × *k*_4_ are the assumed tissue uptake and clearance rate constants, respectively, of the albumin-bound radioligand alone. The factors involving *f*_u_ account for the fact that we describe the system in terms of the total radioligand concentration (free + albumin-bound) in the central compartment (justified by the rapidly established and dynamically maintained equilibrium between both states). Consequently, the tissue uptake rate constants for free and albumin-bound tracer have to be scaled by f_u_ and (1-f_u_), respectively. Likewise, the excretion rate constant has to be scaled by f_u_ due to the general assumption that albumin and thus the albumin-bound ligand are not renally cleared. The conventional assumption that only the free ligand is available for tissue uptake and receptor binding would be described by the setting r_3_ = 0 in this model.

In order to demonstrate the principal effects of different values of f_u_ and r_3_ (the proportional factor by which tissue uptake rate of albumin-bound ligand alone deviates from the uptake rate of free ligand alone), we have computed the explicit solution for the set of differential equations in Figure 6B after impulse input at *t* = 0 yielding the TACs in all three compartments for any chosen set of free parameters. Since the model equation is not amenable to easy analytical solution, we determined the solution by diagonalization of the coefficient matrix of the differential equations via numerical eigenvalue/eigenvector computation. This allows for expressing the impulse response in all compartments as differently weighted sums over three exponentials and is superior in many respects to numerical integration of the differential equation.^41^ Figure 7 shows some representative examples. Here, we chose common values for most parameters—such that the curves are in rough qualitative agreement with our experimental PET data—while only varying *f*_u_ and *r*_3_. Figure 7A shows the results when setting *f*_u_ = 1 and *r*_3_ = 0. This corresponds to the situation where no albumin binding is present at all and serves as the baseline against which to compare the following plots. Figure 7A demonstrates the very rapid decline (on the given time scale of several days) of activity in the central blood pool due to renal excretion and a reversible tissue uptake (receptor binding) of the radioligand leading to a total tumor AUC = 0.67 (in the chosen arbitrary units where *C*_l_(*t* = 0) = *D*/*V* = 1 with D being the injected dose, V being the volume of the central blood pool compartment, reached within about 3 days.

**Figure 7:**
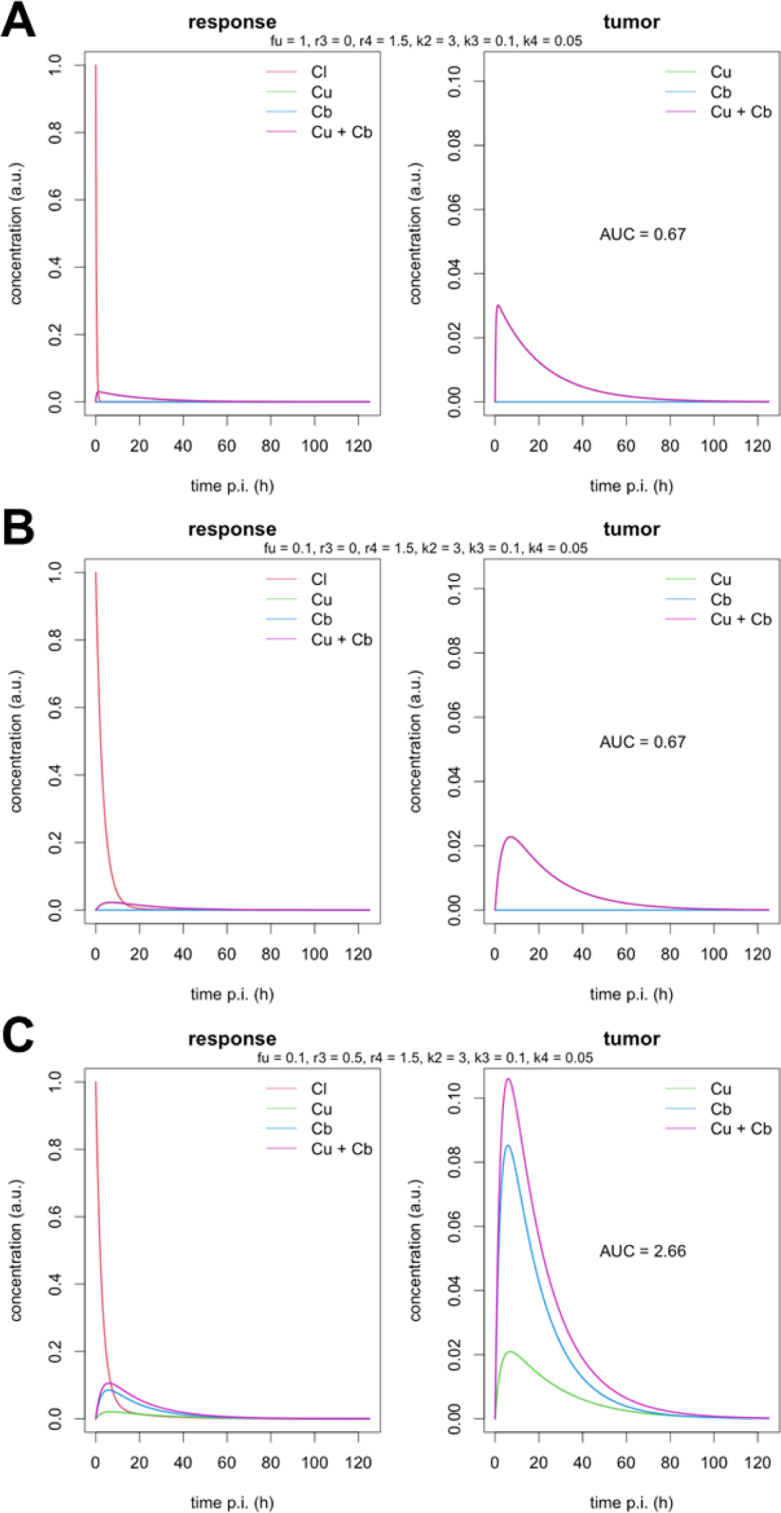
Representative examples of simulated TACs for different situations: (A) ligand has no albumin binding at all (*f*_u_ = 1); (B) ligand with substantial albumin binding (*f*_u_ = 0.1), but tumor uptake is restricted to the free ligand (*r*_3_ = 0); and (C) ligand with substantial albumin binding (*f*_u_ = 0.1) and tumor uptake of the albumin-bound radioligand occurring at half the rate of the free ligand (*r*_3_ = 0.5). TACs were simulated according to the model in Figure 6. Rate constants *k*_2_, *k*_3_, and *k*_4_ are in units of h^–1^. All other parameters are dimensionless. The left-hand plots show the unit impulse response in all three compartments (plus total tissue response) while the right-hand plots omit C_l_ for better discernibility of the tumor response curves.

Figure 7B considers the case *f*_u_ = 0.1 and *r*_3_ = 0 and thus corresponds to a radioligand with substantial albumin binding, while tissue uptake remains restricted to the free ligand. Compared to the situation in Figure 7A, the radioligand’s residence time in the blood pool is distinctly increased (the early-phase excretion rate is reduced by a factor of 10 for *f*_u_ = 0.1), the tumor TAC shape accordingly exhibits a moderate change (broadened and flattened), but the tumor AUC remains completely unaltered. This shows that the frequently voiced explanation that the increase of the radioligand’s residence time in the blood circulation due to albumin binding is the reason for increased integral tumor uptake (AUC) is erroneous. The broadening and flattening of the tumor TAC is in accord with our data for the series of **[** ^**64**^ **Cu]­Cu-NODAGA-cLAB-TATEs** with increasing albumin-binding affinity.^19^

Figure 7C considers the case *f*_u_ = 0.1 and *r*_3_ = 0.5 which amounts to assuming that the albumin-bound ligand undergoes tissue uptake at half the rate of the free radioligand and demonstrates that the tumor AUC is only increasing (and substantially so) if non-negligible tissue uptake of the albumin-bound radioligand is present. The crucial question then is to what extent the noncovalent albumin-radioligand complexes can indeed be transported from the blood circulation to the extravascular space. In fact, several binding receptors are known for transporting native albumin from the blood circulation to the extravascular space.^42–44^ Accordingly, a notable amount of albumin is always present in the extravascular space, albeit at a lower concentration than in the blood circulation: e.g., Ellmerer et al.^45^ determined a mean concentration of 13.25 g/L in human skeletal muscle compared to 48.9 g/L in serum. Consequently, in healthy soft tissue one would expect f_u_ values for our radioligands that are much higher than in the blood pool. However, considering the actually targeted tumor tissue one has to take into account the fact that the usually compromised blood capillary system and reduced lymphatic drainage^18^ could facilitate the extravasation of systemic albumin and increase the extravascular native albumin concentration substantially which in turn would bring the resulting f_u_ in this space in line with the respective value in the blood space. In any case, the proposed compartment model does not account explicitly for possible differences between f_u_ in blood and in tissue and this parameter thus must be interpreted with care, describing summarily the fact that the radioligand is present in two distinct modifications. It might also be noted that even in the improbable case that only free radioligand would reach the extravascular space, albumin-bound radioligand would immediately appear as well due to the presence of albumin in the extravascular space considering the fast binding kinetic to albumin of the albumin-binding moieties. Consequently, albumin-bound radioligand principally should always be able to access (and possibly bind to) the receptor. In the context of our compartment model this fact is accounted for by the uptake rate r_3_ × *k*_3_. It should of course be kept in mind that the used rate constants for tissue uptake and clearance (*k*_3_ and *k*_4_) describe summarily the entire process of uptake and washout, which of course consist of several microscopic processes (e.g., extravasation and receptor binding for tumor uptake). However, this is the standard procedure for PET-compartment modeling approaches.^41^

The overall increase of the tumor AUC relative to the baseline case of Figure 7A is of course dependent on the actually realized model parameters. Focusing on the influence of r_3_ and r_4_ alone, i.e., on the differences between tissue uptake and tissue clearance of the free and albumin-bound ligand, respectively, while keeping all other parameters at their values as used in Figure 7B and Figure 7C, and varying both parameters over a substantial dynamic range the results shown in Figure S18 are obtained.

For completeness, we note that in the above considerations, we have implicitly assumed that the renal excretion rate constant (*k*_2_) and the tissue transport rates (*k*_3_ and *k*_4_) for the free radioligand reflect inherent and invariant properties of the free ligand and did not consider the further possibility that the structural modification of the considered radioligand with albumin-binding moieties itself might alter excretion or tissue uptake rates of the free radioligand relative to the baseline as defined by the unmodified radioligand. If this mechanism were operational and quantitatively relevant, it would, e.g., be conceivable that the modified free radioligand is excreted distinctly slower which would result in a higher AUC of free radioligand in plasma which in turn would then lead to an increase in tumor AUC relative to that observed for the unmodified radioligand. Similarly, an increase of *k*_3_ and/or a reduction of *k*_4_ of the modified free radioligand would increase the tumor AUC as well. While such effects cannot be ruled out completely it seems highly unlikely that they could explain the substantial differences between different compounds as observed in the present investigation where the different compounds exhibit only very subtle structural differences when compared to each other. Specifically, **[** ^**64**^ **Cu]­Cu-NODAGA-cLAB5-TATE** and **[** ^**64**^ **Cu]­Cu-NODAGA-cLAB4-TATE** vary only in the substituent at the phenyl ring of the albumin-binding moiety (F and CH_3_, respectively, Figure 1). Therefore, we consider it much more plausible that the observed large differences in tumor AUC between these two compounds (148 vs 289 SUV mean*h) are caused by additional tumor uptake of albumin-bound ligand than by either a reduced excretion rate or an elevated tumor uptake rate of the free radioligand due to the presence of the albumin-binding moiety *per se*. However, future work will be necessary to clarify the situation definitely.

## Conclusion

The present study adds to our previous characterization of heterobivalent (SST_2_/albumin) radioligands, named **NODAGA-cLAB-TATEs**, with the primary aim of identifying the mechanistic basis for the increased integral tumor uptake of **[** ^**64**^ **Cu]­Cu-NODAGA-cLAB4-TATE** compared to **[** ^**64**^ **Cu]­Cu-NODAGA-TATE**. The *in vivo* characterization of the novel radioligands herein along with the data from our previous study highlight that the increase in tumor uptake does only marginally originate from off-target binding via the albumin-binding moiety itself or retention of the albumin-bound radioligand in the tumor tissue independent of SST_2_-binding. Instead, we propose that the albumin-bound radioligand is able to bind to SST_2_, albeit with a lower affinity compared to the free radioligand, which is deduced from saturation binding analyses in the presence of albumin. Since a clear proof for the binding of the albumin-bound radioligand to SST_2_ is challenging to provide, we sought to quantitatively describe the principal effects of albumin binding of radioligands in the blood circulation on the time course of excretion and tumor uptake using a dedicated three-compartment model. This model strongly suggests that the increase in tumor uptake is not a consequence of the increase in blood circulation time *per se*, which is, however, for some reason often used as the underlying explanation. There must be an additional/changed tumor uptake mechanism to be operational, which fits to our hypothesis for the series of **[** ^**64**^ **Cu]­Cu-NODAGA-cLAB-TATEs** that the fraction of albumin-bound radioligand in addition to the free radioligand is able to bind to the receptor. The capability for the formation of such ternary complexes (albumin-radioligand-target protein) may also apply to albumin-binding radioligands addressing other molecular targets and could explain accordingly their improved integral tumor uptake compared to radioligands without distinct albumin binding. Furthermore, considerations regarding the linker identity and length between the actual targeting unit and the albumin-binding moiety for the prospective design of heterobivalent radioligands appear advisable.

## Experimental Section

### General

All commercial reagents and solvents were used without further purification unless otherwise specified. For the incorporation of NODAGA, the building block (*R*)-NODA-GA­(tBu)_3_ (CheMatech) was used. The synthesis of albumin binders **(** *R* **)-2c** and **(** *S* **)-2d** are described in the Supporting Information. Nuclear magnetic resonance spectra for albumin binders **(** *R* **)-2c** and **(** *S* **)-2d** were recorded on an Agilent Technologies 400 MR spectrometer consisting of 400/54 premium compact magnet, 400 MR console and 400 MHz OneNMRProbe PT probe head (400 MHz for ^1^H, 101 MHz for ^13^C and 376 MHz for ^19^F). Spectra were processed by using the program MestreNova (version 14.2.1–27684). NMR chemical shifts were referenced to the residual solvent resonances relative to tetramethylsilane (TMS; ^1^H and ^13^C) and trichlorofluoromethane (CFCl_3_; ^19^F). Mass spectra (ESI) were obtained on a Waters Xevo TQ-S mass spectrometer driven by the Mass Lynx software. All regression analyses were done with GraphPad Prism (version 10.4.1, GraphPad Software, San Diego, CA, USA). The purity of the albumin binders **(** *R* **)-2c** and **(** *S* **)-2d** and the peptides **NODAGA-cLAB­(5–6)-TATEs** and **NODAGA-cLAB4-** *scr* **TATE** proved to be ≥95% as analyzed by analytical RP-HPLC. DOTA-EB-TATE was kindly provided by Dr. Xiaoyuan (Shawn) Chen from the National University of Singapore.

### Chromatography

The HPLC system used was a LC-20A Prominence HPLC by Shimadzu, consisting of degasser unit DGU-20A5R, two separate pumping units LC-A20R, sample manager SIC-20ACHT, column oven CTO-20AC, PDA-detector SPD-M20A, communication-bus module CBM-20A and fraction collector FRC-10A. Two Aeris Peptide 5 μm XB-C18 columns (100 Å, 250 × 4.6 mm and 250 × 21.2 mm) were used as the stationary phases for analytical and preparative RP-HPLC, respectively. A binary gradient system of 0.1% CF_3_COOH/water (solvent A) and 0.1% CF_3_COOH/CH_3_CN (solvent B) at a flow rate of 1 mL/min (analytical) or between 10 and 20 mL/min (preparative) served as the eluent. High-resolution mass spectra (HRMS) were obtained on a Q-TOF MS using electrospray ionization: Agilent 1260 Infinity II HPLC (Santa Clara, California, USA; pump G7111B, autosampler G7129A, column oven G7116N, UV detector G7717C, eluent MeCN/water acidified with 0.1% formic acid 80/20, bypass mode) coupled to UHD Accurate Mass Q-TOF LC MS G6538A. The measurements were performed in bypass mode using an eluent consisting of (A) 0.1% formic acid in CH_3_CN and (B) 0.1% formic acid in H_2_O; flow rate, 0.25 mL/min. A reference mass solution containing ammonium trifluoroacetate, hexakis­(1*H*,1*H*,3*H*-tetrafluoropropoxy)­phosphazene, and purine was continuously coinjected via a dual ESI source. For UPLC-DAD-MS, a system from Waters (ACQUITY UPLC I class system including an ACQUITY UPLC PDA e λ detector coupled to a Xevo TQ-S mass spectrometer) was used. An ACQUITY UPLC BEH C18 column (1.7 μm, 130 Å, 100 × 2.1 mm, equipped with a ACQUITY UPLC BEH C18 VanGuard Precolumn, 1.7 μm, 130 Å, 5 × 2.1 mm) was used as a stationary phase. A binary gradient system of 0.1% CH_3_COOH/water (solvent A) and 0.1% CH_3_COOH in CH_3_CN/CH_3_OH (1:1, v/v, solvent B) at a flow rate of 0.4 mL/min served as the eluent. Analytical radio-HPLC was performed on a Series 1200 device (Agilent Technologies, Santa Clara, CA, USA) equipped with a Ramona β/γ-ray detector (Raytest, Straubenhardt, Germany). Eluent A: 0.1% (v/v) trifluoroacetic acid in H_2_O; eluent B: 0.1% (v/v) trifluoroacetic acid in acetonitrile; HPLC system: Zorbax SB-C18, 300 Å, 4 μm, 250 × 9.4 mm (Agilent); gradient elution using 95% eluent A for 5 min, 95% eluent A to 95% eluent B in 10 min, 95% eluent B for 5 min and 95% eluent B to 95% eluent A in 5 min, 3 mL/min, 50 °C, recovery of activity (decay-corrected) was >95%. For the plasma stability measurements, a modified gradient system was used: Gradient elution using 65% eluent A for 5 min, 65% eluent A to 85% eluent B in 10 min, 85% eluent B for 5 min and 85% eluent B to 65% eluent A in 5 min, 3 mL/min, 50 °C.

### General Solid-Phase Syntheses of Peptides

The TATE derivatives were synthesized as previously described.^19,27^ Briefly, Wang resin preloaded with Fmoc-Thr­(*t*Bu)–OH was used as the starting material, and the further amino acids were introduced by repetitive cycles of Fmoc removal (20% piperidine/DMF) and coupling (HATU/DIPEA/DMF) using an automated microwave peptide synthesizer (Initiator+ Alstra from Biotage). Differently, (*R*)-NODA-GA­(tBu)_3_ was manually coupled. The albumin binders were coupled by on-resin CuAAC (CuSO_4_/THPTA/sodium ascorbate). Cleavage from the resin and concomitant removal of all protecting groups was realized by treatment with TFA/H_2_O/TIPS (95/2.5/2.5), and after removal of TFA, the peptides were precipitated with ice-cold diethyl ether. For disulfide formation, the peptides were dissolved in DMSO/CH_3_CN/H_2_O (1/2.5/6.5, adjusted to pH 8.0 with 1% NH_3_). For **NODAGA-cLAB6-TATE**, cyclization was performed on resin with iodine in DMF. The crude peptides were purified by RP-HPLC.

### Radiolabeling and Radiopharmacological In Vitro Characterization of DOTA/NODAGA-Bearing Peptides

#### Radiolabeling

[^64^Cu]­CuCl_2_ was produced at the Helmholtz-Zentrum Dresden-Rossendorf on the 30 MeV TR-Flex-cyclotron (Advanced Cyclotron Systems Inc., ACSI, Canada) by a ^64^Ni­(p,n)^64^Cu nuclear reaction as reported previously.^46,47^ Radiolabeling of the peptides herein was performed as previously described for **NODAGA-cLAB-TATEs** or **NODAGA-NES-TATEs**.^19,27^ Briefly, peptides (6 nmol, stock solution in DMSO) were labeled with [^64^Cu]­CuCl_2_ (100–250 MBq) in ammonium acetate buffer (pH 5.5, 20 min, 60–80 °C). Labeling yields were usually ≥98% and apparent molar activities (molar activities calculated based on the applied peptide amount, no separation of the unlabeled peptide was conducted after radiolabeling) between 17 and 40 GBq/μmol were achieved. The radioligand stock solution (0.2 M NH_4_OAc, 1 MBq/μL, 50 μM) was diluted with PBS (10 mM, pH 7.4) or 0.154 M NaCl for further experiments.

#### Ultrafiltration Assay

The ultrafiltration assay used to quantify the binding of the ^64^Cu-labeled peptides to HSA was performed as previously described for **NODAGA-cLAB-TATEs**.^19,27^ Briefly, a constant activity amount of the radioligands (10–15 MBq) were mixed with different HSA concentrations (200 nM–1.4 mM). Aliquots were loaded into the ultrafiltration devices (4104 centrifugal filter units by Millipore; 30 000 Da nominal molecular weight limit, methylcellulose micropartition membranes) and centrifuged. After centrifugation, aliquots from the top and bottom fractions were measured in a γ-counter (PerkinElmer, Wizard^2^ 2480 automatic gamma counter). The retained fraction was calculated as a quotient of these measurements. The dissociation constant, *K*_d_, was calculated using the Morrison equation.

#### *N*-Octanol/PBS Distribution Coefficient (log D_7.4_ Value)

The determination of the log D_7.4_ value was done in triplicate as previously described.^19,27^ Briefly, a sample of the radioligand was added to the 1:1 mixture of PBS/n-octanol. The radioactivity in a defined volume of each layer was measured (ISOMED 2100). The distribution coefficient was expressed as the logarithm of the ratio of counts per minute (cpm) measured in the n-octanol phase to the cpm measured in the PBS phase.

#### Plasma Stability Assay

For human plasma, venous blood (4.5 mL) from one of two healthy, male volunteers who were not fasting or on any medication was collected. Blood samples were dispensed into S-Monovette (Sarstedt) plasma separator tubes containing lithium heparin-coated particles. The tubes were allowed to stand on ice for 30 min protected from light followed by centrifugation at 2000 rcf for 10 min at 4 °C. Samples were visually checked for hemolysis and interference, stored at 4 °C (protected from light) and used within a week. For mouse plasma, arterial blood (>1 mL) from a NMRI-nu/nu mouse was obtained by heart puncture. Blood samples were dispensed into lithium heparin (heparin-sodium LEO 25,000 IU/5 mL)-flushed 1.5 mL Eppendorf tubes. The tubes were allowed to stand on ice for 30 min protected from light followed by centrifugation at 2000 rcf for 10 min at 4 °C. Samples were visually checked for hemolysis and interference and used at the same day.

Plasma stability of the radioligands was done as previously described for **NODAGA-cLAB-TATE** and **NODAGA-NES-TATEs**.^19,27^ Briefly, an aliquot of the radioligand was added to plasma and stability was assessed at different time points by withdrawing an aliquot, followed by protein precipitation and analysis by radio-HPLC.

#### In vitro SST_2_ Binding Affinity Using Intact MPC Cells

Binding assay was performed as previously described.^27,31^ A number of 3 × 10^5^ cells/cm^2^ were seeded in collagen-coated 48-well microplates (CELLSTAR 48 Well Cell Culture Multiwell Plates, Polystyrene, Greiner Bio-One, item no. 677180) and grown for 3 days. For binding assays, the cell culture medium was removed and replaced by fresh medium supplemented with the radioligand (Am = 25 MBq/nmol) at increasing final concentrations between 0.321 and 40 nM (final sample volume: 0.2 mL). Nonspecific cell binding was measured in the presence of nonlabeled DOTA-TATE or Acetyl-TATE at a final concentration of 1 μM. Nonspecific binding to plastic surfaces was determined in cell-free wells at radioligand concentrations of 0.312, 1.25, 5, and 20 nM (with and without 1 μM nonlabeled DOTA-TATE or Acetyl-TATE; the values at the other radioligand concentrations were derived by linear regression). Samples were incubated for 60 min at 37 °C. Incubation was stopped by washing with ice-cold Dulbecco’s PBS. Cells were lysed with 0.1 M NaOH containing 1% (w/v) SDS. Activity was measured in cell homogenates and in a series of radioligand standards containing increasing molar amounts between 0.06 and 8 pmol using the gamma counter Wizard (PerkinElmer). The protein content of cell homogenates was measured as described above. Measurements for total binding were performed in triplicate, while measurements for nonspecific binding were performed in duplicates.

For saturation binding of **[** ^**64**^ **Cu]­Cu-NODAGA-cLAB2-TATE** in the presence of varying HSA concentrations, HSA (from 10% solution in 0.85% NaCl and 0.05% NaN_3_) at final concentrations of 0, 1, 5, 20, and 40 μM was added to the medium supplemented with the radioligand prior to the addition to all wells (total binding, nonspecific cell binding, nonspecific plastic binding). For 0–5 μM HSA, radioligand concentrations between 0.321 and 40 nM were used, for 20 μM HSA between 0.625 and 80 nM and for 40 μM HSA between 1.25 and 160 nM. All other steps were performed as described above.

#### Determination of K_d_ and *B*_max_ Values

Calculations were performed as previously described.^27,31^ Plots of “total binding” = *f*(radioligand) were analyzed by nonlinear regressions using the model of “one site-total, accounting for ligand depletion” as implemented in GraphPad Prism and plots of “nonspecific binding” = *f*(radioligand) were analyzed by linear regressions (Figure S3). For the ligand depletion model, the term “NS” was constrained to the respective slopes obtained by the linear regressions. The terms “SpecAct” (obtained with standard curves, Figure S4) and “Vol” (0.2 mL, assay volume) were also constrained. *K*_d_ values were derived in nM, and the *B*_max_ values (in cpm) were transformed into fmol/mg. Both data sets were corrected for nonspecific binding, i.e., binding to the microplate cavities (in the absence of intact cells).

#### Schild analysis^38^

For Schild analysis, the ratio (similar to the agonist dose ratio, DR) between the apparent *K*_d_ value in the presence of each HSA concentration and the *K*_d_ value in the absence of HSA was calculated. Then, the logarithm of these DR minus 1 (log­(DR-1)) was calculated and plotted against the log of the concentration of HSA. This plot was analyzed by linear regression with the *x*-intercept equals to the log­(*K*_d_) value of the radioligand for HSA.

#### Cell Binding and Uptake

Cell binding and uptake were measured as previously described.^27,31^ MPC cells were seeded into collagen-coated 24-well microplates and cultivated for 4 days. All washing steps were performed using PBS containing 0.9 mM CaCl_2_ and 0.5 mM MgCl_2_. Total radioligand uptake was measured after incubation with the radioligand (*A*_m_ = 30 GBq/μmol) at a final concentration of 20 nM in RPMI 1640 medium with GlutaMAX supplement (Thermo Fisher Scientific) for 1 h at 37 and 4 °C. Nonspecific binding was determined in the presence of 20 μM Acetyl-TATE. The uptake fraction was measured after acid wash of cell surface-bound radioligand with wash buffer containing 0.05 M glycine, pH 2.8, for 5 min. The activity of cell homogenates was measured using the γ counter Wizard (PerkinElmer). The protein content of cell homogenates was measured as described above.

### Experimental Animals and PET/CT imaging^19^

All animal experiments were performed following the protocols evaluated and approved by the Landesdirektion Sachsen, Referat 25 - Veterinärwesen, Lebensmittelüberwachung and Pharmazie (09105 Chemnitz, Germany, ethics approval number: 25-5131/562/52).

A number of 4 × 10^6^ MPC cells were resuspended in 40 μL of Dulbecco’s phosphate-buffered saline and injected subcutaneously into the shoulder of 7–10-week-old female nude mice (Rj:NMRI-Foxn1^nu/nu^, Janvier Laboratories, Le Genest-Saint-Isle, France). General anesthesia was induced and maintained with inhalation of 10% (v/v) desflurane in 30% oxygen/air (v/v). Tumor growth was monitored using caliper measurements. Animals were sacrificed using CO_2_ inhalation and cervical dislocation. When diameters of MPC tumors reached 8 ± 3 mm (volumes of 65–282 mm^3^), small-animal positron emission tomography (PET) was performed using the nanoScan PET/CT (Mediso Medical Imaging Systems, Budapest, Hungary) and Si78 PET/CT (BRUKER, Billerica, MA) instruments. Scanners were cross-calibrated using a syringe source containing 10 MBq of [^64^Cu]­CuCl_2_ diluted in 2 mL of 0.01 M HCl. Each mouse (*n* = 2) received between 7 and 10 MBq of the respective radioligand (*A*_m_ = 40 GBq/μmoL) delivered in 0.154 M NaCl via intravenous injection through a tail vein catheter within the initial 30 s after scan start. A series of PET/CT scans were performed at defined time points after radioligand injection (0–2 h [dynamically], 5 h [4.5–5.5 h], 24 h [23–25 h], and 48 h [46–50 h]). Standardized uptake values (SUV = [MBq detected activity/mL tissue]/[MBq injected activity/g body weight]) were determined in defined volumes of interest (VOIs) and reported as VOI-averaged SUV_mean_ ± range [min–max]. Assuming a density of 1 g/mL for body tissues, the SUVs become dimensionless. Image recording, image reconstruction, and data analysis were performed as reported previously.^19^

## Supplementary Material





## Notes

HSA binding curves for ^64^Cu-labeled **NODAGA-cLAB4-scrTATE**, **NODAGA-cLAB­(5/6)-TATE**, and **DOTA-EB-TATE**; radio-HPLC chromatograms of **[** ^**64**^ **Cu]­Cu-NODAGA-cLAB5-TATE**, **[** ^**64**^ **Cu]­Cu-NODAGA-cLAB6-TATE**, and **[** ^**64**^ **Cu]­Cu-NODAGA-cLAB-** *scr* **TATE** upon incubation in mouse and human plasma; exemplary saturation binding and standard curves of ^64^Cu-labeled **NODAGA-cLAB4-scrTATE**, **NODAGA-cLAB­(5/6)-TATE**, and **DOTA-EB-TATE** using intact MPC cells; cell binding data of ^64^Cu-labeled **NODAGA-cLAB4-scrTATE**, **NODAGA-cLAB­(5/6)-TATE**, and **DOTA-EB-TATE**; time-activity curves of liver and muscle uptake for ^64^Cu-labeled **NODAGA-cLAB4-scrTATE**, **NODAGA-cLAB­(5/6)-TATE**, and **DOTA-EB-TATE**; side-by-side comparison of calculated AUC_0–48h_ values and tumor-to-organ ratios of **[** ^**64**^ **Cu]­Cu-NODAGA-TATE**, **[** ^**64**^ **Cu]­Cu-NODAGA-Pra-PEG2-TATE**, and **[** ^**64**^ **Cu]­Cu-NODAGA-cLAB5-TATE**; side-by-side comparison of time-activity curves of tumor and heart uptake for **[** ^**64**^ **Cu]­Cu-NODAGA-cLAB4-TATE**, **[** ^**64**^ **Cu]­Cu-NODAGA-cLAB5-TATE**, and **[** ^**64**^ **Cu]­Cu-NODAGA-cLAB6-TATE**; side-by-side comparison of calculated AUC_0–48h_ values and the tumor-to-organ ratios of **[** ^**64**^ **Cu]­Cu-NODAGA-cLAB4-TATE** and **[** ^**64**^ **Cu]­Cu-NODAGA-cLAB6-TATE**; cellular release of **[** ^**64**^ **Cu]­Cu-NODAGA-cLAB4-TATE** and **[** ^**64**^ **Cu]­Cu-NODAGA-cLAB6-TATE**; comparison of parameters for tumor and kidney uptake for the **[** ^**64**^ **Cu]­Cu-NODAGA-cLAB-TATEs**; relationship between AUC_0–48h_ (heart) and the albumin-binding affinity for the series of **[** ^**64**^ **Cu]­Cu-NODAGA-cLAB-TATEs** and **[** ^**64**^ **Cu]­Cu-cLAB4-** *scr* **TATE**; side-by-side comparison of time-activity curves of tumor and heart uptake for **[** ^**64**^ **Cu]­Cu-NODAGA-cLAB2-TATE** and **[** ^**64**^ **Cu]­Cu-DOTA-EB-TATE**; side-by-side comparison of **[** ^**64**^ **Cu]­Cu-NODAGA-cLAB4-TATE** and **[** ^**64**^ **Cu]­Cu-DOTA-EB-TATE**; exemplary saturation binding curves for **[** ^**64**^ **Cu]­Cu-NODAGA-cLAB2-TATE** toward SST_2_ at different HSA concentrations using intact MPC cells; exemplary saturation binding curves for **[** ^**64**^ **Cu]­Cu-NODAGA-TATE** toward SST_2_ at different HSA concentrations using intact MPC cells; graphical depiction of *K*_d_[SST_2_] and *B*_max_ values from saturation binding analyses for **[** ^**64**^ **Cu]­Cu-NODAGA-cLAB2-TATE** depending on the present HSA concentration; influence of r_3_ and r_4_ on the AUC boost factor; comparison of estimated and determined *K*_d_[HSA] values for the ^64^Cu-labeled TATE derivatives bearing an albumin binder; compound characterization data­(PDF)
Molecular formular strings (CSV)

## Abbreviations used

AUC: area under curve
cLAB: “clickable” lysine-derived albumin binders;
EB: Evans Blue
HSA: human serum albumin
PET: positron emission tomography
PPB: plasma protein binding
SST_2_: somatostatin receptor subtype 2
SUV: standardized uptake value.
