# Supplementary material for: Deciphering the Tumor Uptake of Heterobivalent (SST2/Albumin) [64Cu]Cu-NODAGA-cLAB-TATEs
Source: J Med Chem. 2025 May 20;68(11):12029–46. doi: 10.1021/acs.jmedchem.5c00890 (PMC12169663; doi:10.1021/acs.jmedchem.5c00890)
Supplement: Supplementary file 1 [file jm5c00890_si_001.pdf]

Supporting Information

Deciphering the tumor uptake of heterobivalent (SST_2/albumin) [^{64}Cu]Cu-NODAGA-cLAB-TATEs

Florian Brandt[a,b,c,#], Martin Ullrich[a,#], Markus Laube[a], Reik Löser[a,c], Jörg Kotzerke[b], Klaus Kopka[a,c,d,e], Jens Pietzsch[a,c], Jörg van den Hoff[a,c], Robert Wodtke[a,*]

[a] Helmholtz-Zentrum Dresden-Rossendorf, Institute of Radiopharmaceutical Cancer Research, Bautzner Landstraße 400, 01328 Dresden, Germany

[b] University Hospital Carl Gustav Carus at the Technische Universität Dresden, Klinik und Poliklinik für Nuklearmedizin, Fetscherstraße 74, 01307 Dresden, Germany

[c] Technische Universität Dresden, Faculty of Chemistry and Food Chemistry, Mommsenstraße 4, 01069 Dresden, Germany

[d] German Cancer Consortium (DKTK), Partner Site Dresden, 01307 Dresden, Germany

[e] National Center for Tumor Diseases (NCT), Partner Site Dresden, University Cancer Center (UCC), 01307 Dresden, Germany

# These authors contributed equally to this work.

*E-mail: r.wodtke@hzdr.de

Table of Contents

Figure S1: **HSA binding curves** **3**

Figure S2: **Plasma stability** **4**

[^{64}Cu]Cu-NODAGA-cLAB5-TATE 4

[^{64}Cu]Cu-NODAGA-cLAB6-TATE 5

[^{64}Cu]Cu-NODAGA-cLAB4-*scr*TATE 6

Figure S3: **Exemplary saturation binding curves using intact MPC cells** **7**

Figure S4: **Exemplary standard curves** **8**

Figure S5: **Cell binding data** **9**

Figure S6: **Time-activity curves of liver and muscle uptake for [^{64}Cu]Cu-NODAGA-cLAB5-TATE (A), [^{64}Cu]Cu-NODAGA-cLAB6-TATE (B), [^{64}Cu]Cu-NODAGA-cLAB4-*scr*TATE (C), and [^{64}Cu]Cu-DOTA-EB-TATE (D)** **10**

Figure S7: **Side-by-side comparison of calculated $AUC_{0\text{-}48h}$ values (A) and the tumor-to-organ ratios (B) of [^{64}Cu]Cu-NODAGA-TATE, [^{64}Cu]Cu-NODAGA-Pra-PEG2-TATE, and [^{64}Cu]Cu-NODAGA-cLAB5-TATE** **11**

Figure S8: **Side-by-side comparison of time-activity curves of tumor (A) and heart uptake (B) for [^{64}Cu]Cu-NODAGA-cLAB4-TATE, [^{64}Cu]Cu-NODAGA-cLAB5-TATE, and [^{64}Cu]Cu-NODAGA-cLAB6-TATE** **12**

Figure S9: **Side-by-side comparison of calculated $AUC_{0\text{-}48h}$ values (A) and the tumor-to-organ ratios (B) of [^{64}Cu]Cu-NODAGA-cLAB4-TATE and [^{64}Cu]Cu-NODAGA-cLAB6-TATE** **13**

Figure S10: **Cellular release of [^{64}Cu]Cu-NODAGA-cLAB4-TATE and [^{64}Cu]Cu-NODAGA-cLAB6-TATE** **14**

Figure S11: **Comparison of parameters for tumor and kidney uptake for the [^{64}Cu]Cu-NODAGA-cLAB-TATEs** **15**

Figure S12: **Relationship between $AUC_{0\text{–}48h}$[heart] and the albumin-binding affinity for the series of [^{64}Cu]Cu-NODAGA-cLAB-TATEs and [^{64}Cu]Cu-cLAB4-*scr*TATE** **16**

Figure S13: **Side-by-side comparison of time-activity curves of tumor (A) and heart uptake (B) for [^{64}Cu]Cu-NODAGA-cLAB2-TATE and [^{64}Cu]Cu-DOTA-EB-TATE** **17**

Figure S14: **Side-by-side comparison of [^{64}Cu]Cu-NODAGA-cLAB4-TATE and [^{64}Cu]Cu-DOTA-EB-TATE** **18**

Figure S15: **Exemplary saturation binding curves for [^{64}Cu]Cu-NODAGA-cLAB2-TATE toward SST_2 at different HSA concentrations using intact MPC cells** **19**

Figure S16: **Exemplary saturation binding curves for [^{64}Cu]Cu-NODAGA-TATE towards SST_2 at different HSA concentrations using intact MPC cells** **20**

Figure S17: **Graphical depiction of $K_d[SST_2]$ and B_{max} values from saturation binding analyses for [^{64}Cu]Cu-NODAGA-cLAB2-TATE depending on the present HSA concentration** **21**

Figure S18: **Influence of r_3 and r_4 on the AUC boost factor** **22**

Table S1: **Comparison of estimated and determined K_d[HSA] values for the ^{64}Cu-labeled TATE derivatives bearing an albumin binder** **23**

Compound characterization data **24**

Final albumin binders 24

TATE derivatives 27

^{64}Cu-labeled TATE derivatives 30

References **33**

Figure S1: HSA binding curves

Plots of “percentage of retained activity” = f([HSA]) with nonlinear regressions using eq I (Morrison equation). Data shown are mean values of two (±SD) single experiments. The concentration of the radioligands was adjusted to 2 µM (**[^{64}Cu]Cu-DOTA-EB-TATE**) or 20 µM (all other radioligands). Dotted lines indicate 0, 50, and 90% of retained activity. Compound names were abbreviated.

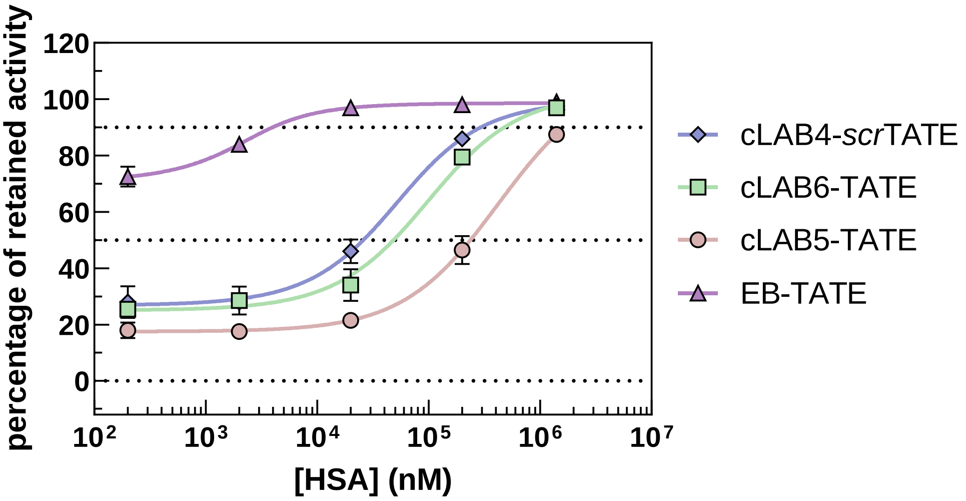

Figure S2: Plasma stability

Radio-HPLC chromatograms of **[^{64}Cu]Cu-NODAGA-cLAB5-TATE**, **[^{64}Cu]Cu-NODAGA-cLAB6-TATE**, and **[^{64}Cu]Cu-NODAGA-cLAB-*scr*TATE** after ^{64}Cu-labeling and incubation in human and mouse plasma for 24 h. For **[^{64}Cu]Cu-NODAGA-cLAB5-TATE** and **[^{64}Cu]Cu-NODAGA-cLAB-*scr*TATE**, incubations in mouse plasma were also performed after preincubation with *bis*-para-nitrophenylphosphate (BNPP, 300 µM, 60 min) to show that the observed transformation for both radioligands is caused by plasma carboxylesterases.

[^{64}Cu]Cu-NODAGA-cLAB5-TATE

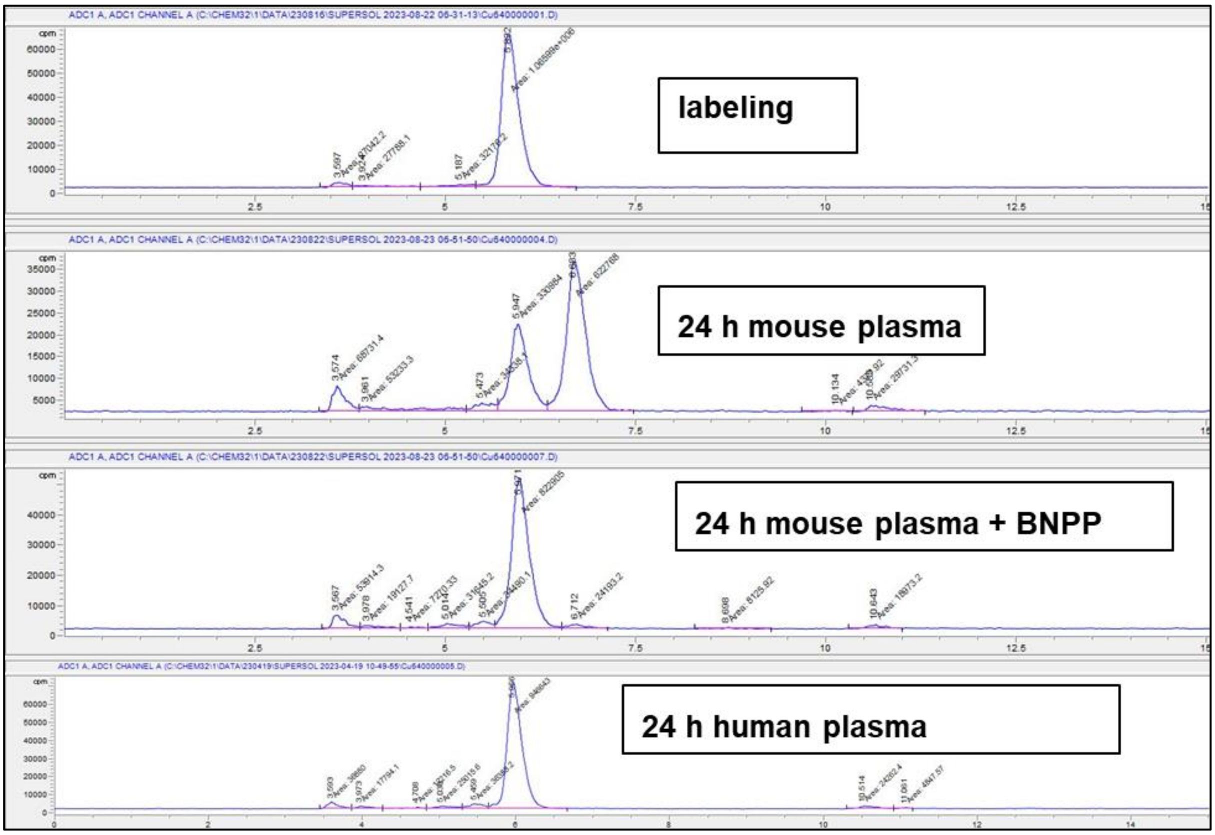

[^{64}Cu]Cu-NODAGA-cLAB6-TATE

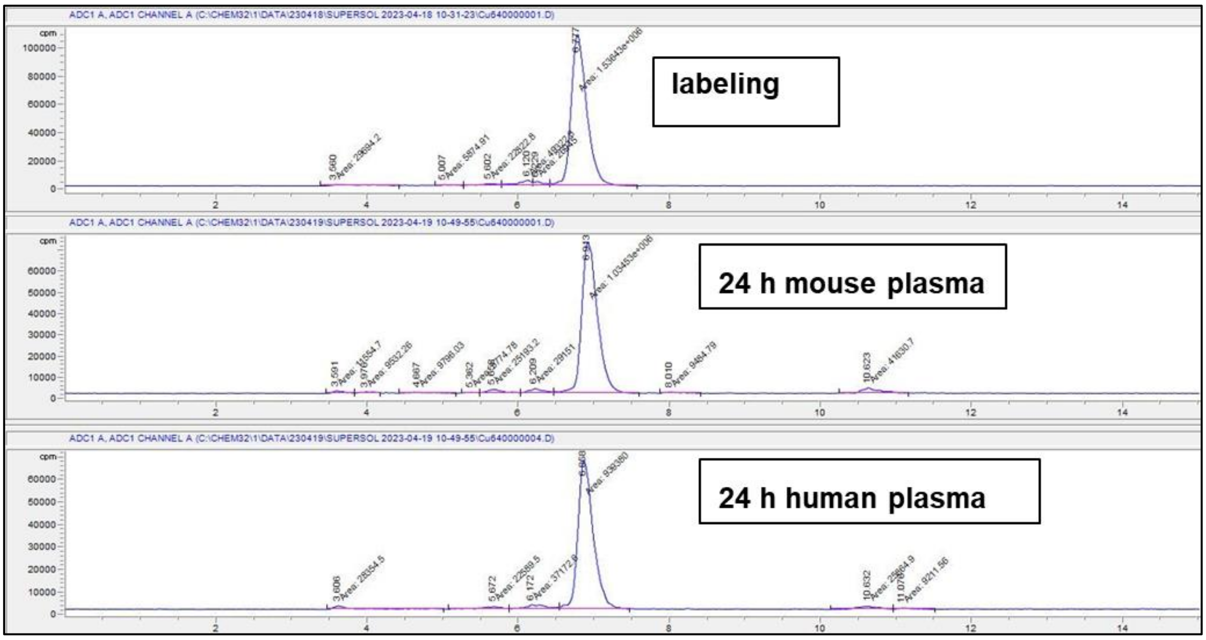

[^{64}Cu]Cu-NODAGA-cLAB4-*scr*TATE

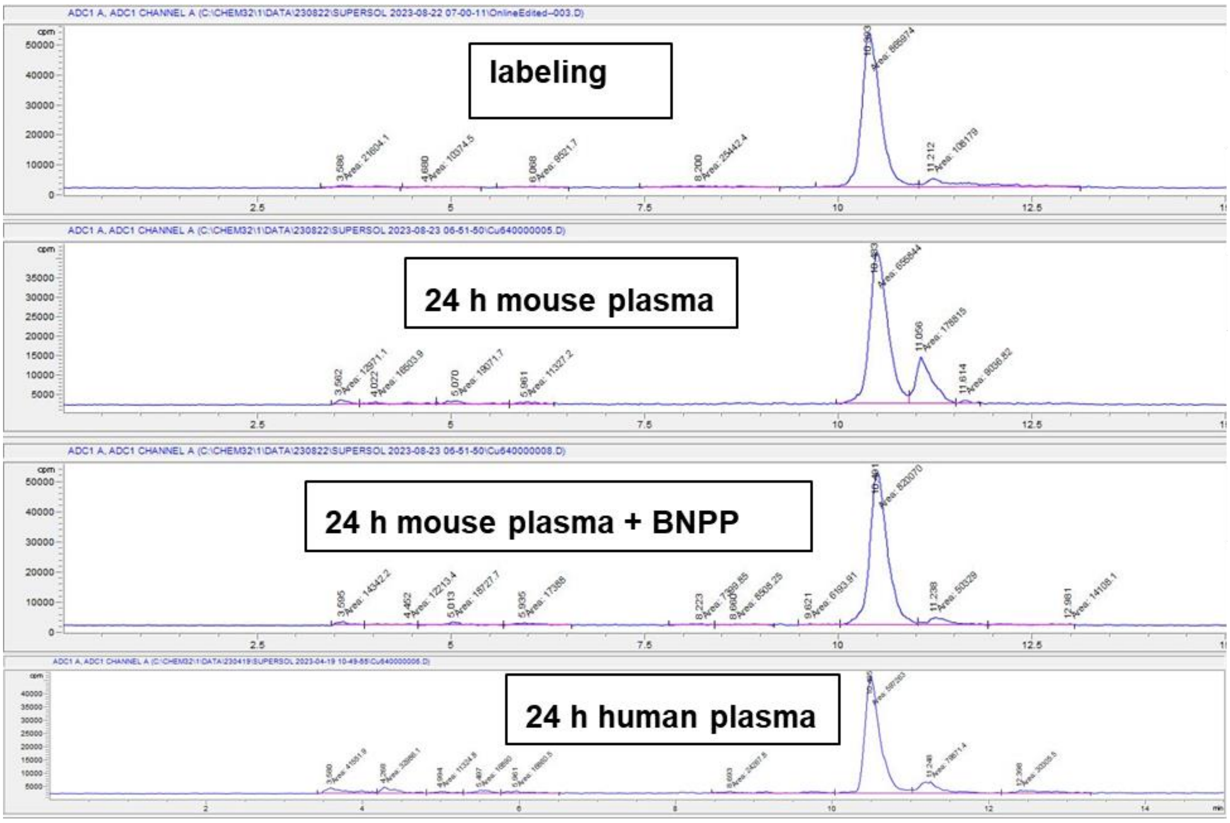

Figure S3: Exemplary saturation binding curves using intact MPC cells

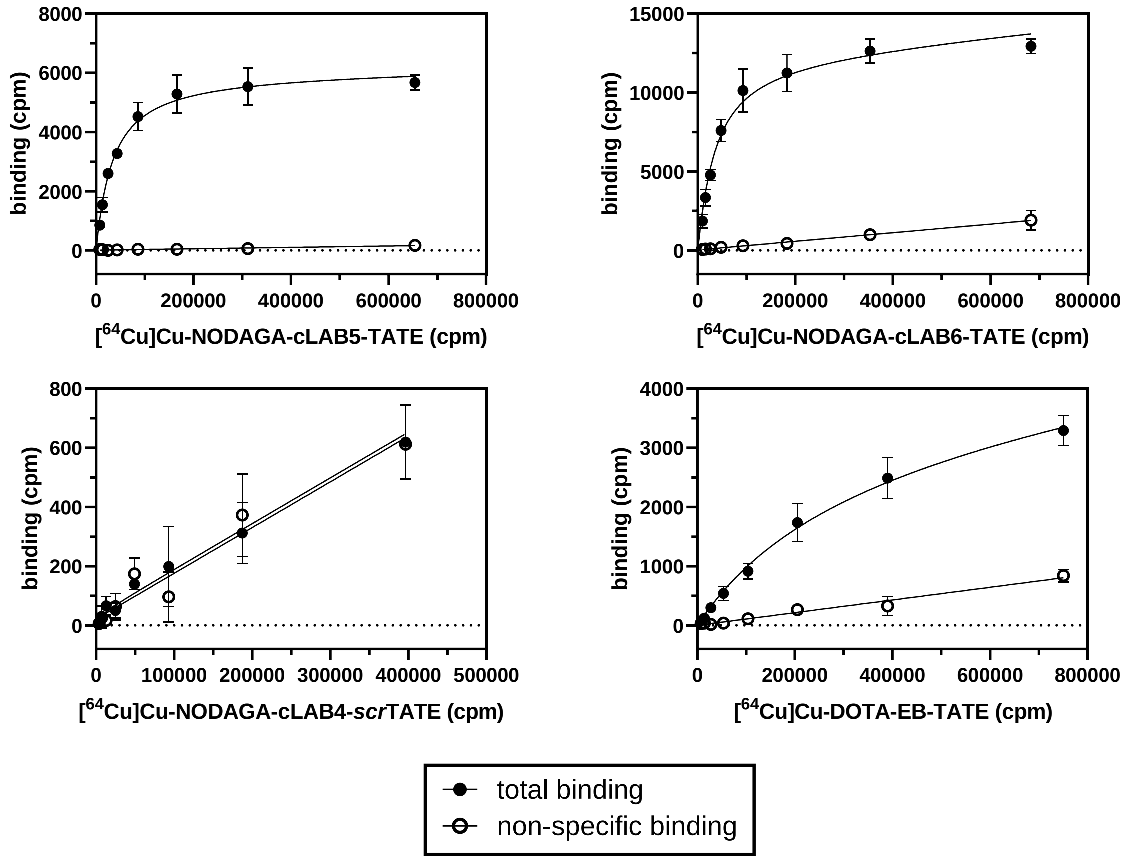

Figure S4: Exemplary standard curves

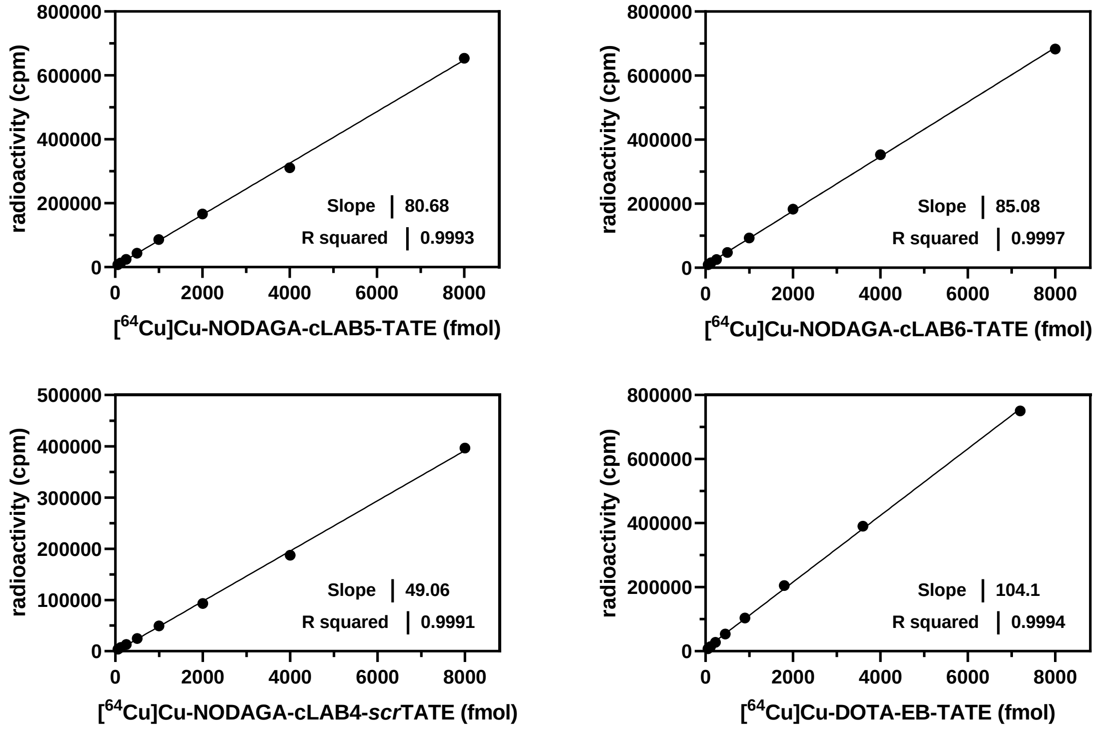

Figure S5: Cell binding data

Data shown in **A** and **B** are mean values (±SD) of one experiment (**NODAGA-cLAB(5/6)-TATE** and **NODAGA-cLAB4-*scr*TATE**) or 3 experiments (±SEM, **DOTA-EB-TATE**), which were performed in sextuplicate. In **C**, the amount of specific “internalized” (acid resistant) radioligand (red bars in **A**, mean values) is expressed as percentage of specific total-bound radioligand (blue bars in **A**, mean values). The difference to 100% was assigned to “surface-bound” radioligand. For a better overview, the compound names were abbreviated.

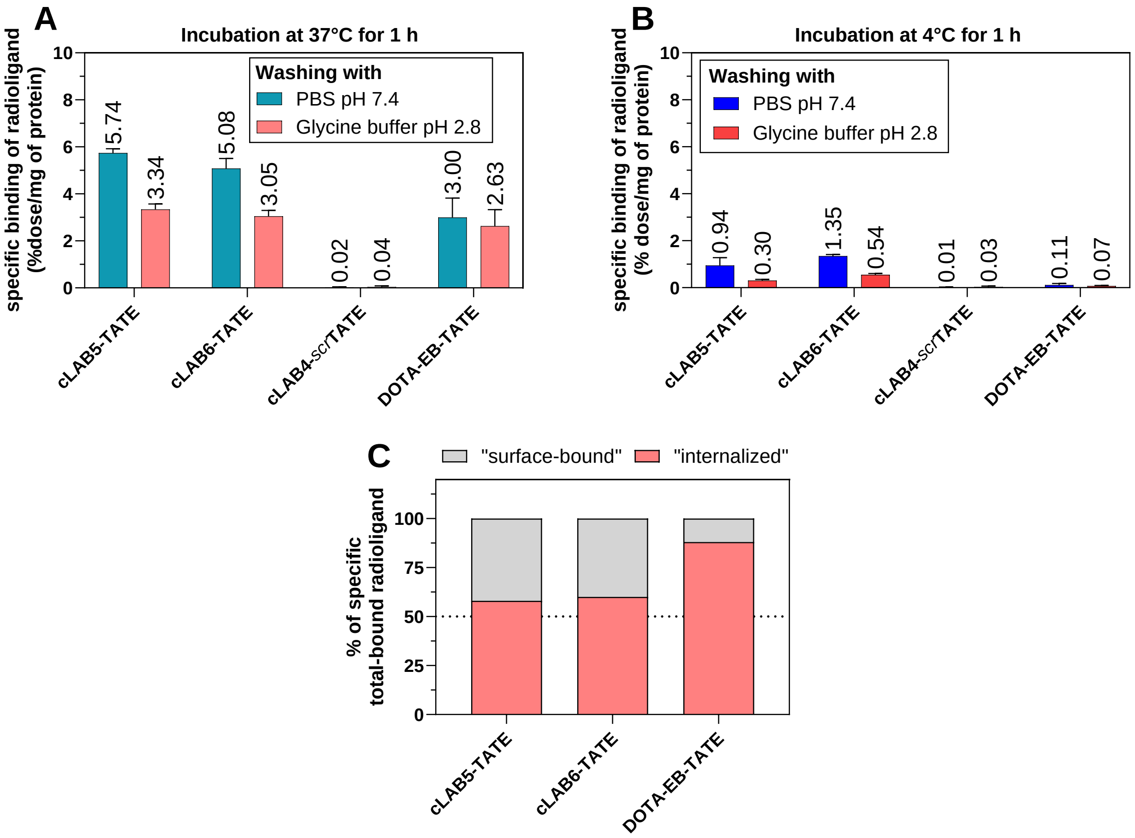

Figure S6: Time-activity curves of liver and muscle uptake for [^{64}Cu]Cu-NODAGA-cLAB5-TATE (A), [^{64}Cu]Cu-NODAGA-cLAB6-TATE (B), [^{64}Cu]Cu-NODAGA-cLAB4-*scr*TATE (C), and [^{64}Cu]Cu-DOTA-EB-TATE (D)

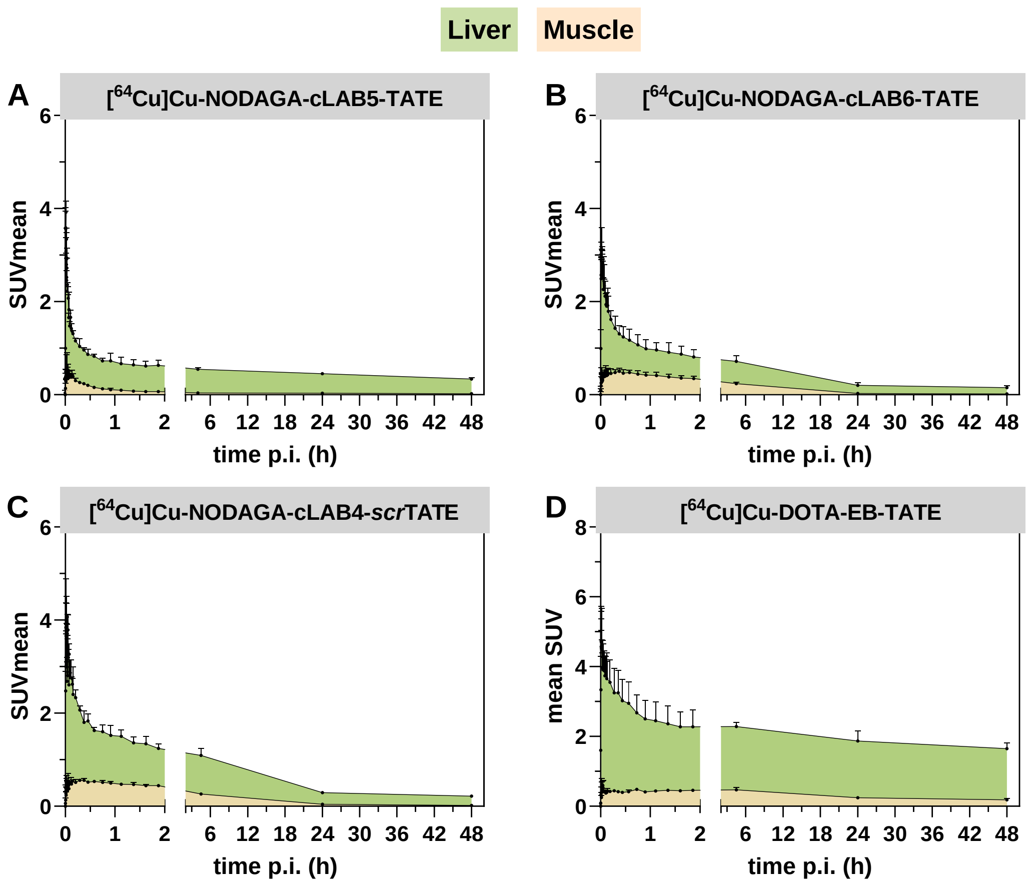

Figure S7: Side-by-side comparison of calculated $AUC_{0\text{-}48h}$ values (A) and the tumor-to-organ ratios (B) of [^{64}Cu]Cu-NODAGA-TATE, [^{64}Cu]Cu-NODAGA-Pra-PEG2-TATE, and [^{64}Cu]Cu-NODAGA-cLAB5-TATE

Calculated $AUC_{0\text{-}48h}$ values and the tumor-to-organ ratios for the different ^{64}Cu-labeled TATE derivatives are shown in **A** and **B**, respectively. Data for **[^{64}Cu]Cu-NODAGA-TATE** and **[^{64}Cu]Cu-NODAGA-Pra-PEG2-TATE** were reproduced from reference [1]. Copyright 2022 American Chemical Society.

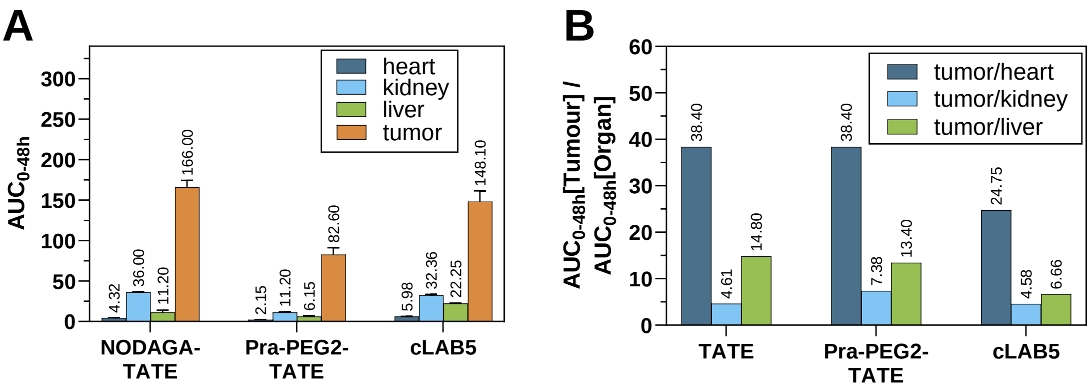

Figure S8: Side-by-side comparison of time-activity curves of tumor (A) and heart uptake (B) for [^{64}Cu]Cu-NODAGA-cLAB4-TATE, [^{64}Cu]Cu-NODAGA-cLAB5-TATE, and [^{64}Cu]Cu-NODAGA-cLAB6-TATE

Data for **[^{64}Cu]Cu-NODAGA-cLAB4-TATE** were reproduced from reference [1]. Copyright 2022 American Chemical Society.

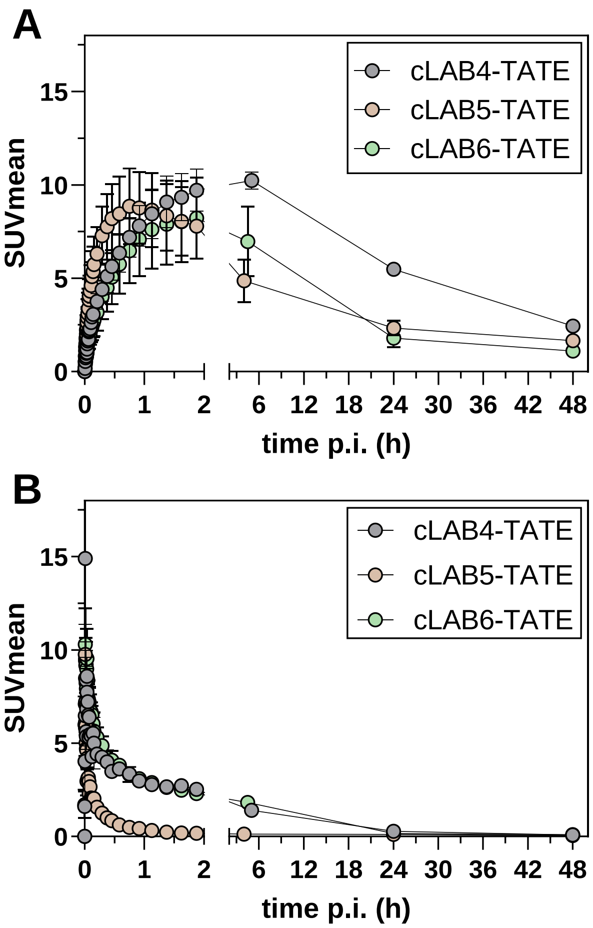

Figure S9: Side-by-side comparison of calculated $AUC_{0\text{-}48h}$ values (A) and the tumor-to-organ ratios (B) of [^{64}Cu]Cu-NODAGA-cLAB4-TATE and [^{64}Cu]Cu-NODAGA-cLAB6-TATE

Data for **[^{64}Cu]Cu-NODAGA-cLAB4-TATE** were reproduced from reference [1]. Copyright 2022 American Chemical Society.

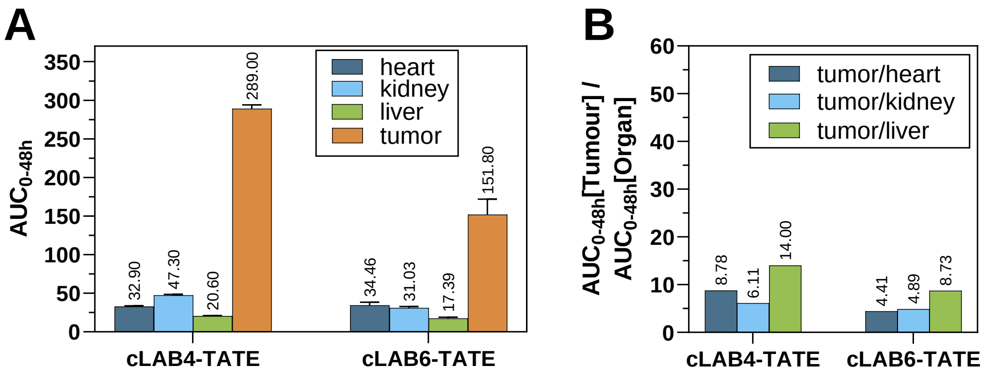

Figure S10: Cellular release of [^{64}Cu]Cu-NODAGA-cLAB4-TATE and [^{64}Cu]Cu-NODAGA-cLAB6-TATE

Activity release from intact MPC cells after incubation at a radioligand concentration of 25 nM followed by radioligand removal; cells were incubated with radioligands for 1 hour at 37° C; non-specific binding was determined in presence of 1 µmol/L acetyl-TATE.[2] Data shown are mean values (±SEM) of 4 (**cLAB4-TATE**) or 2 (**cLAB6-TATE**) experiments, which were performed in sextuplicate. Data for **[^{64}Cu]Cu-NODAGA-cLAB4-TATE** were reproduced with permission from reference [2]. Copyright 2024 Ivyspring International Publisher. Data were analyzed by nonlinear regression according to two phase decay. Half-lives of 0.5 and 1 h for the fast fraction and 20 and 12.3 h for the slow fractions were derived for **cLAB4-TATE** and **cLAB6-TATE**, respectively.

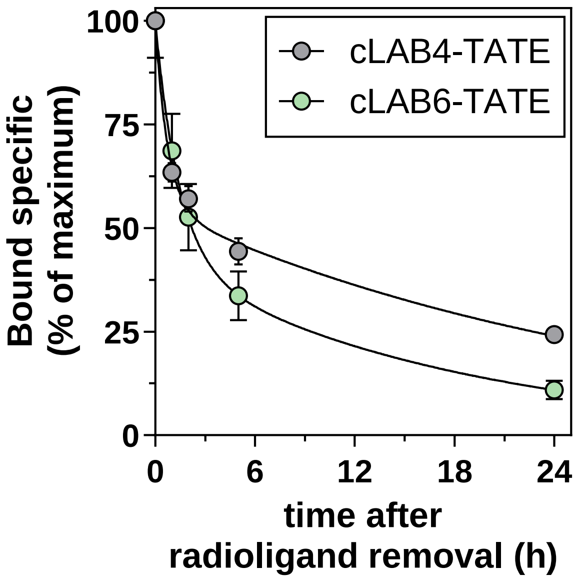

Figure S11: Comparison of parameters for tumor and kidney uptake for the [^{64}Cu]Cu-NODAGA-cLAB-TATEs

A) Sorting of **NODAGA-cLAB-TATEs** in ascending order of their albumin-binding affinity. The fraction of unbound radioligand, f_u, was calculated using the equation $f_u = K_d/(K_d+B_{max})^3$ and assuming a albumin concentration of 500 µM in the blood circulation. **B/C**) Plots of different parameters for tumor (**B**) and kidney (**C**) uptake as a function of the albumin-binding affinity. The dotted lines correspond to the respective values of $AUC_{0\text{-}48\ h}$, SUVmean Peak and SUVmean 48 h *p.i.* of **[^{64}Cu]Cu-NODAGA-TATE**.

A

radioligand	K_d(HSA) in µM	fu
cLAB2	1.8	0.004
cLAB1	3.1	0.006
cLAB3	9.2	0.018
cLAB4	50	0.091
cLAB6	88	0.150
cLAB5	440	0.468

B

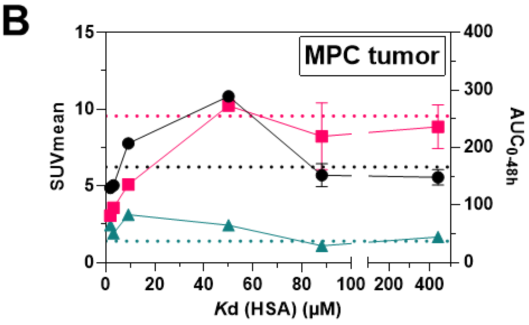

C

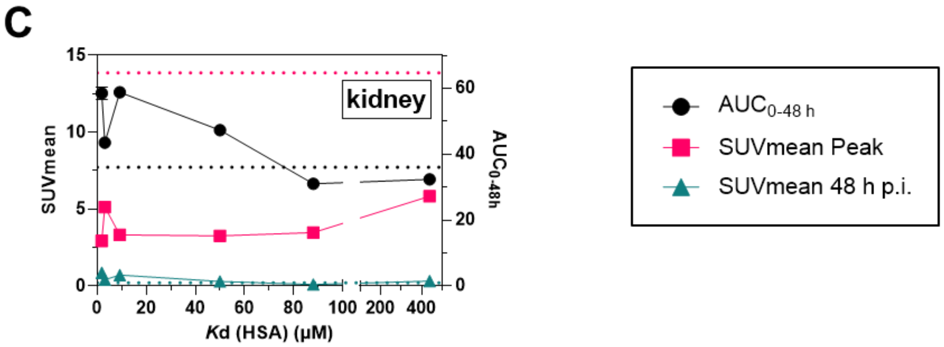

Figure S12: Relationship between $AUC_{0–48h}$[heart] and the albumin-binding affinity for the series of [^{64}Cu]Cu-NODAGA-cLAB-TATEs and [^{64}Cu]Cu-cLAB4-*scr*TATE

Plots of log($AUC_{0–48\,h}$[heart]) = f(log(K_d[HSA])) using the mean values of AUC and K_d[HSA] (in nM) for **[^{64}Cu]Cu-NODAGA-cLAB(1−6)-TATE** and **[^{64}Cu]Cu-NODAGA-cLAB4-*scr*TATE**. Regression analysis was performed by linear regression; n denotes the amount of data points, s denotes the standard deviation of the regression equation, and R^2 denotes the coefficient of determination.

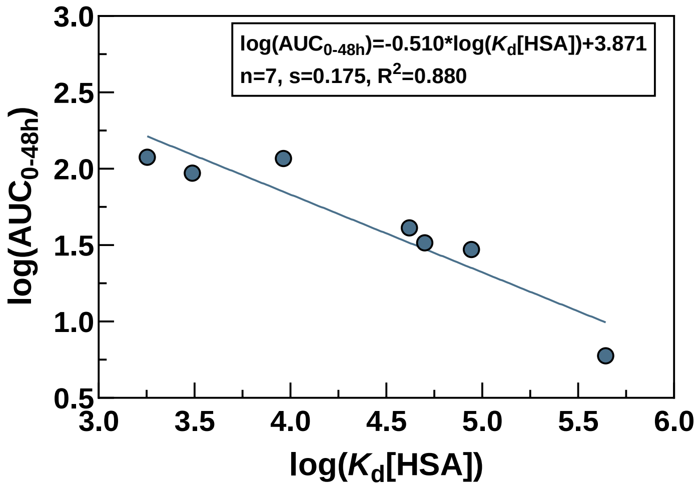

Figure S13: Side-by-side comparison of time-activity curves of tumor (A) and heart uptake (B) for [^{64}Cu]Cu-NODAGA-cLAB2-TATE and [^{64}Cu]Cu-DOTA-EB-TATE

Data for **[^{64}Cu]Cu-NODAGA-cLAB2-TATE** were reproduced from reference [1]. Copyright 2022 American Chemical Society.

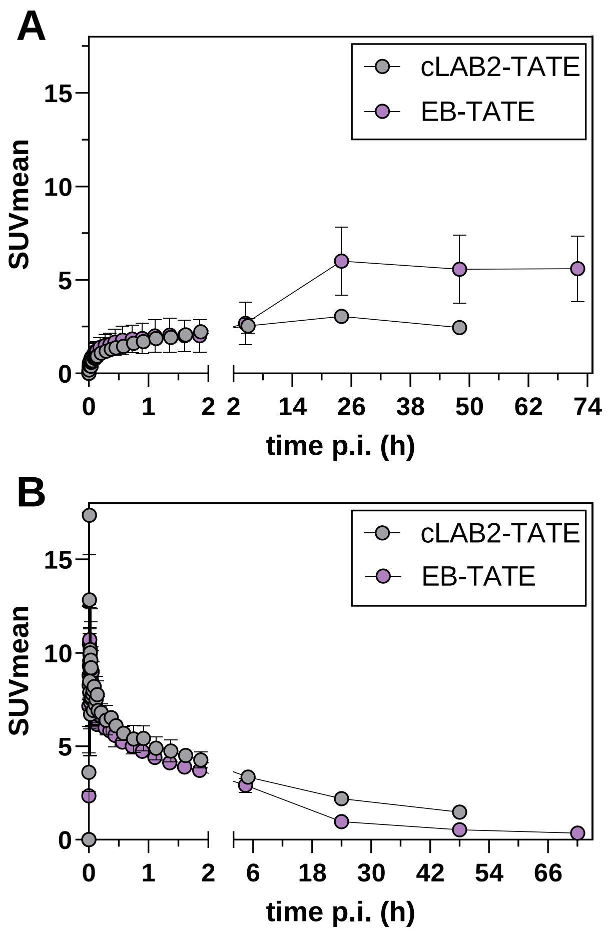

Figure S14: Side-by-side comparison of [^{64}Cu]Cu-NODAGA-cLAB4-TATE and [^{64}Cu]Cu-DOTA-EB-TATE

Both compounds are compared regarding time-activity curves of tumor (**A**), heart (**B**), and kidney (**C**) uptake as well as $AUC_{0\text{-}48h}$ (**D**) and tumor-to-organ ratios (**E**). Data for **[^{64}Cu]Cu-NODAGA-cLAB4-TATE** were reproduced from reference [1]. Copyright 2022 American Chemical Society.

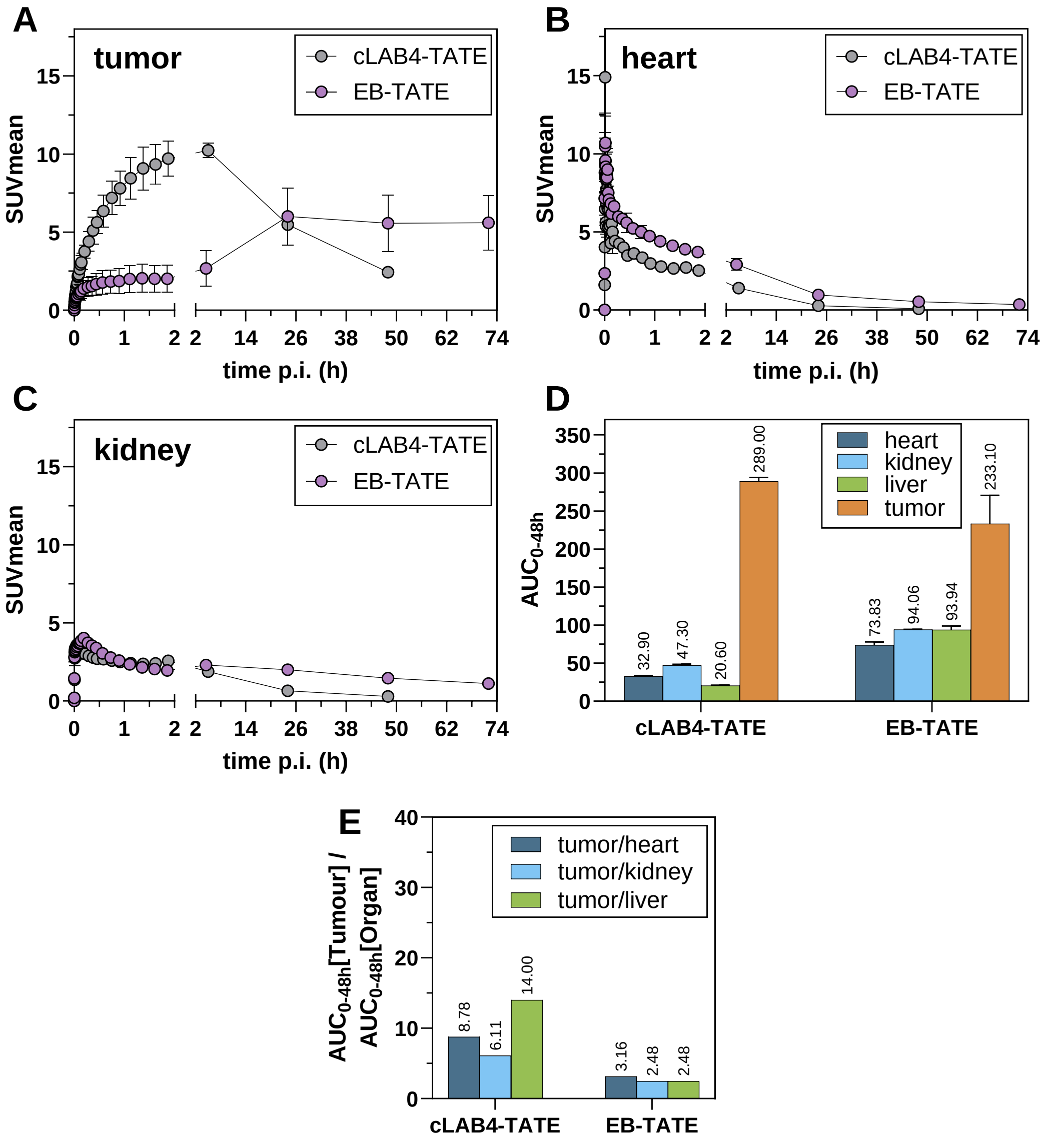

Figure S15: Exemplary saturation binding curves for [^{64}Cu]Cu-NODAGA-cLAB2-TATE toward SST$_2$ at different HSA concentrations using intact MPC cells

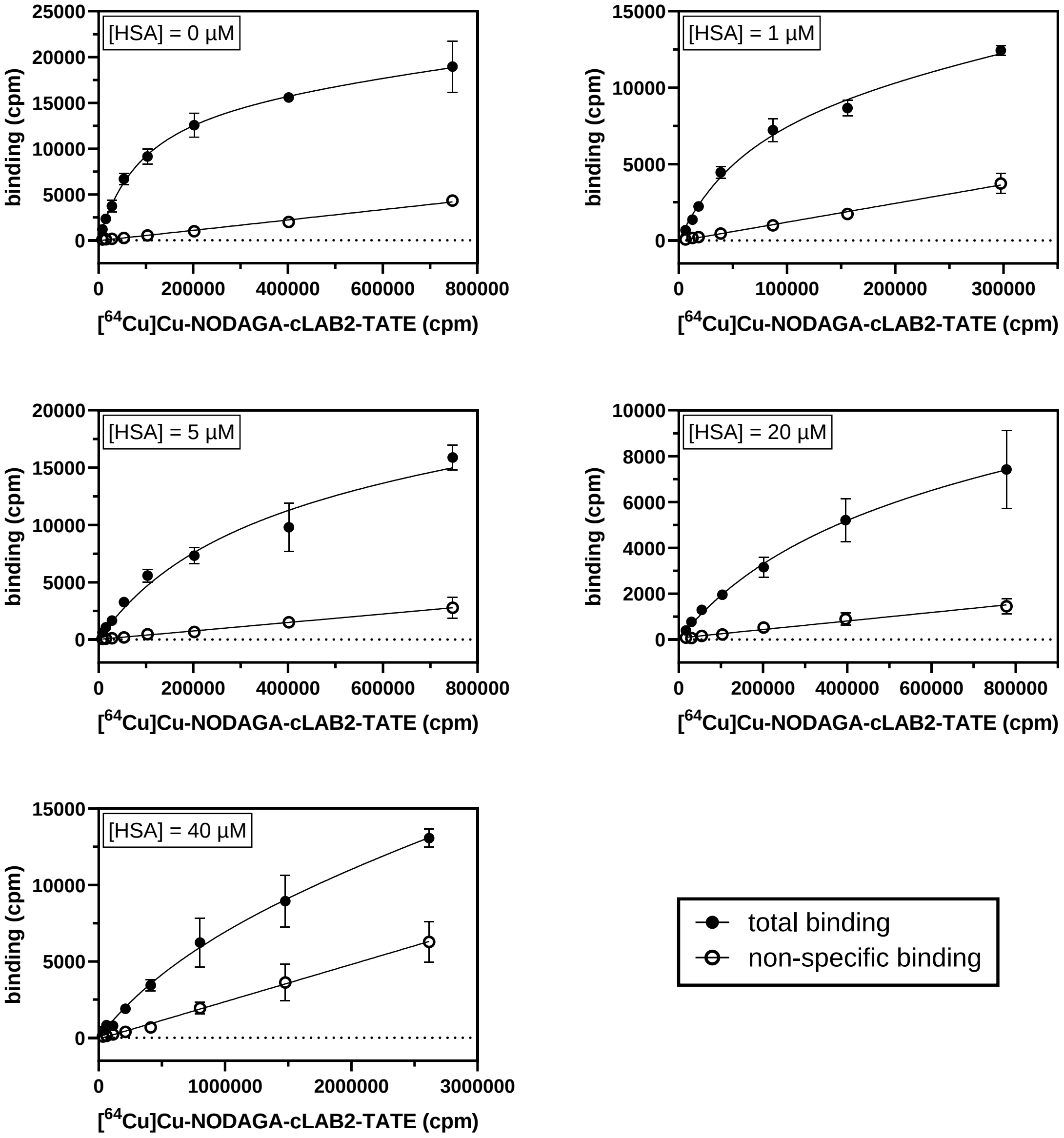

Figure S16: Exemplary saturation binding curves for [^{64}Cu]Cu-NODAGA-TATE towards SST_2 at different HSA concentrations using intact MPC cells

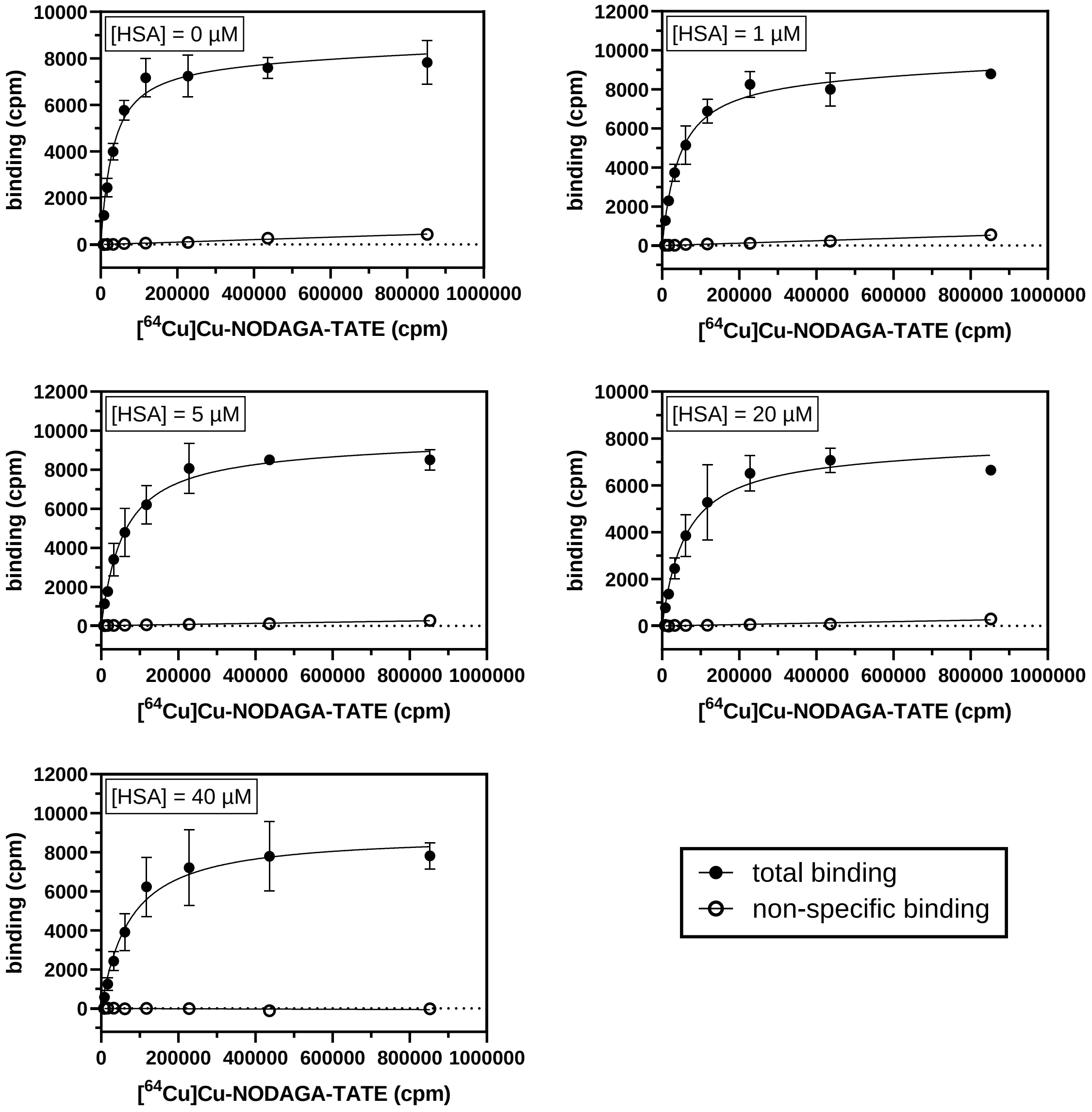

Figure S17: Graphical depiction of K_d[SST_2] and B_{max} values from saturation binding analyses for [^{64}Cu]Cu-NODAGA-cLAB2-TATE depending on the present HSA concentration

While there is no clear trend visible for the B_{max} values, the K_d values seem to approach a certain threshold value.

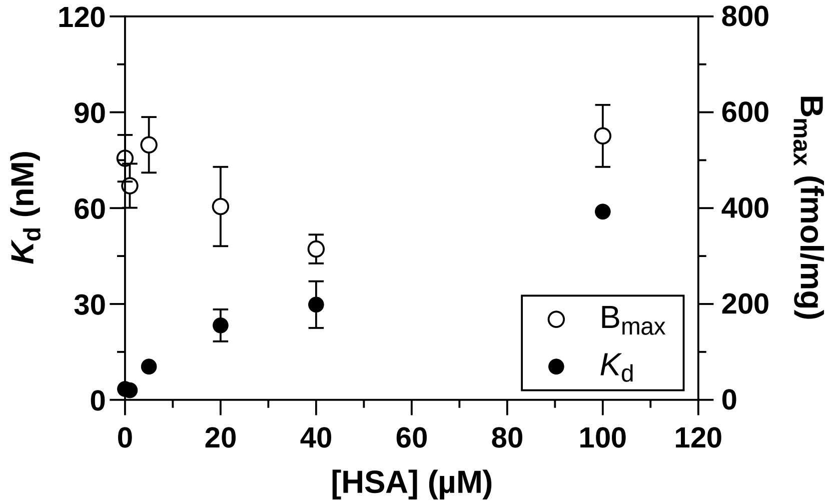

Figure S18: Influence of r_3 and r_4 on the AUC boost factor

The image shows the “AUC boost factor” by which the tumor AUC is increased relative to the r_3=0 case for any given combination of r_3 and r_4 in the considered range. For instance, the point (r_3=0.5, r_4=1.5) corresponds to the AUC ratio between Figure 7B and C in the main article. As this image demonstrates again, an AUC increase is only observed for r_3>0. The boost factor increases with increasing r_3 (higher tissue uptake of albumin-bound ligand) and decreases with increasing r_4 (higher tissue clearance of albumin-bound ligand) as should be expected.

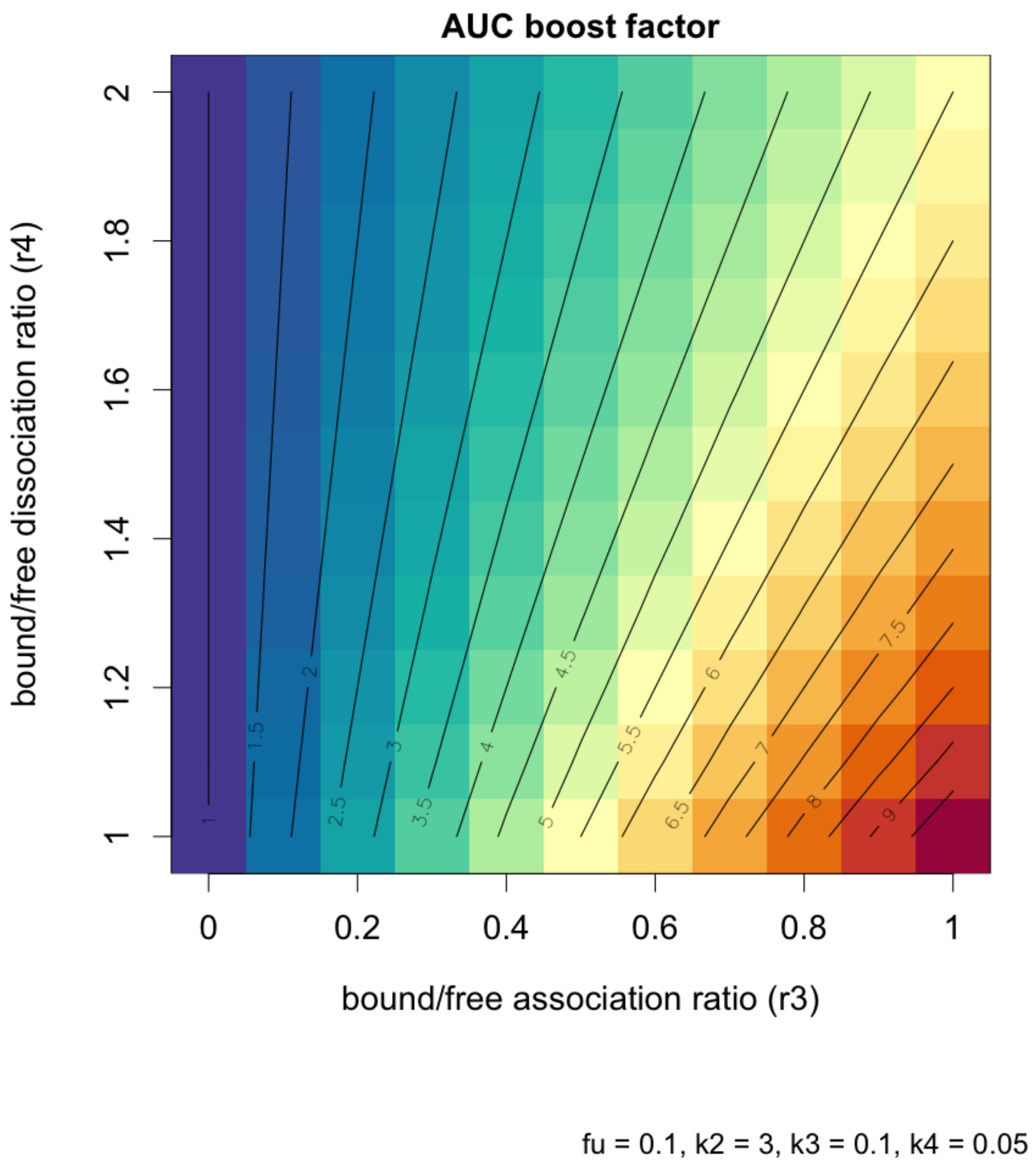

Table S1: Comparison of estimated and determined K_d[HSA] values for the ^{64}Cu-labeled TATE derivatives bearing an albumin binder

compound[h]	albumin binder	K_d[HSA] (µM) predicted	K_d[HSA] (µM) determined[i]	Ratio K_d predict./determ.
**cLAB1**	R^1-pentanoyl-D-Lys(IPB)-OH	8.0[a]	3.1[j]	2.6
**cLAB2**	R^1-benzoyl-L-Lys(IPB)-OH	0.36[b]	1.8[j]	0.2
**cLAB3**	R^1-benzoyl-L-Lys(IPB)-NH_2	7.9[c]	9.2[j]	0.9
**cLAB4**	R^1-benzoyl-L-Lys(MPB)-NH_2	66[d]	50[j]	1.3
**cLAB5**	R^1-benzoyl-L-Lys(FPB)-NH_2	572[e]	440	1.3
**cLAB6**	R^1-benzoyl-D-Lys(MPB)-NH_2	132[f]	88	1.5
**cLAB4-*scr*TATE**	R^1-benzoyl-L-Lys(MPB)-NH_2	66[d]	42	1.6

[a] Represents the K_d value of the albumin binder **(*R*)-1e** (5-N_3-pentanoyl-D-Lys(IPB)-OH).[1]
[b] Represents the K_d value of the albumin binder **(*S*)-1f** (4-N_3-benzoyl-L-Lys(IPB)-OH).[1]
[c] Calculated using the K_d value of the respective carboxylic acid (**(*S*)-1f**) and a factor of 22, which is the factor between Acetyl-L-Lys(IPB)-OH (**(*S*)-1c**) an Acetyl-L-Lys(IPB)-NH_2 (**(*S*)-2a**).[1]
[d] Calculated using the K_d value of the respective carboxylic acid (**(*S*)-1g**, 3.0 µM) and a factor of 22 (carboxylic acid → carboxamide).[1]
[e] Calculated using the K_d value of the respective carboxylic acid (**(*S*)-1i**, 26 µM) and a factor of 22 (carboxylic acid → carboxamide).[1]
[f] Calculated using the K_d value of the respective carboxylic acid (**(*S*)-1f**) and the factors of 22 (carboxylic acid → carboxamide) and 2.2 (L → D configuration, factor between **(*S*)-1c** and **(*R*)-1c**).[1]
[g] Represents the K_d value determined for the non-radioactive compound using the fluorescence-based competition assay.[1]
[h] Compound names were abbreviated.
[i] Determined using the radiometric ultrafiltration assay described in the main article.
[j] Previously determined by us.[1]

Compound characterization data

All HPLC methods are encoded as follows:

$X\_g_{start}\text{-}g_{end}\_t_{iso}\_t_{grad}\_f\_t_{total}$

x x = H for HPLC

g_{start} concentration eluent B between t_0 and start of gradient

g_{End} concentration of eluent B at the end of gradient

t_{iso} time point when gradient starts (suffix: iso)

t_{grad} duration gradient in minutes (suffix: min)

f flow in mL/min (suffix:mL)

t_{total} duration of each run including wash and equilibration phase (suffix: total)

Final albumin binders

***N^{α}*-(4-Azidobenzoyl)-*N^{ε}*-(4-(4-fluorophenyl)butanoyl)-L-lysine amide ((*S*)-2d)**

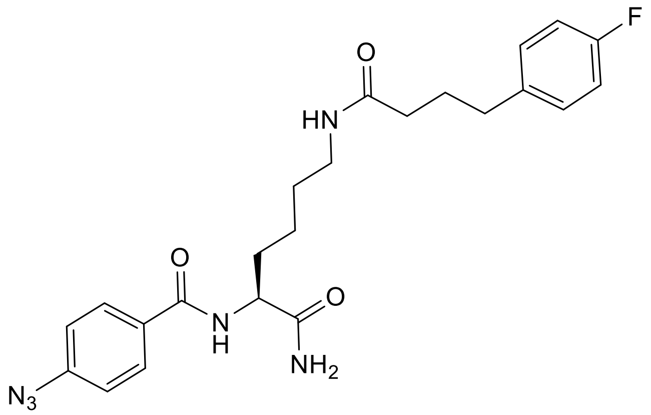

H_25-75_5iso_25min_1mL_42total t_R= 22.0 min

Compound **(*S*)-2d** (43 mg, 23% overall yield, slightly yellow solid) was synthesized starting from Fmoc-L-Lys(Boc)-OH according to the optimized synthetic strategy for carboxamides of lysine-based albumin binders (Scheme 3 in ref [1]. **^{1}H-NMR** (DMSO-d_6) δ = 8.32 (d, ^3J=8.0 Hz, 1H, $N_{\alpha}H$), 7.95 (d, ^3J=8.9 Hz, 2H, H-2,6 azidobenzoyl), 7.75 (t, ^3J=5.7 Hz, 1H, $N_{\varepsilon}H$), 7.38 (s, 1H, N*H*H), 7.22-7.14 (m, 4H, H-2,6 fluorophenyl and H-3,5 azidobenzoyl), 7.07 (t, ^3J=8.9 Hz, 2H, H-3,5 fluorophenyl), 6.98 (s, 1H, NH*H*), 4.33 (td, ^3J=8.7 Hz, 1H, $C_{\alpha}H$ lysine), 3.01 (m, 2H, $C_{\varepsilon}H_2$), 2.02 (t, ^3J=7.4 Hz, 2H, $C_{\alpha}H_2$ butanoyl), 1.82–1.64 (m, 4H, $C_{\beta}H_2$ lysine and $C_{\beta}H_2$

butanoyl), 1.46–1.19 (m, 4H, $C_{\delta}H_2$ and $C_{\gamma}H_2$ lysine). The signal for $C_{\gamma}H_2$ butanoyl overlaps with the solvent signal. **^{13}C-NMR** (DMSO-d_6) δ = 173.90 (C=O), 171.54 (C=O), 165.32 (C=O), 160.56 (d, $^1J_{C,F}$=240.9 Hz, C-4 fluorophenyl), 142.25 (C-4 azidobenzoyl), 137.84 (d, $^4J_{C,F}$=3.1 Hz, C-1 fluorophenyl), 130.77 (C-1 azidobenzoyl), 129.97 (d, $^3J_{C,F}$=7.8 Hz, C-2,6 fluorophenyl), 129.42 (C-2,6 azidobenzoyl), 118.71 (C-3,5 azidobenzoyl), 114.86 (d, $^2J_{C,F}$=20.8 Hz, C-3,5 fluorophenyl), 53.24 (C_{α} lysine), 38.21 (C_{ε} lysine), 34.71 (C_{α} butanoyl), 33.73 (C_{γ} butanoyl), 31.26 (C_{β} lysine), 28.89 (C_{δ} lysine), 27.10 (C_{β} butanoyl), 23.23 (C_{γ} lysine); **^{19}F-NMR** (DMSO-d_6) δ = -117.79 (tt, $^3J_{H,F}$=9.0 Hz, $^4J_{H,F}$=5.5 Hz; fluorophenyl). MS (ESI$^+$): m/z calculated for $C_{23}H_{27}FN_6O_3$: 455.22 $[M+H]^+$, found: 455.8.

***N*$^{\alpha}$-(4-Azidobenzoyl)-*N*$^{\varepsilon}$-(4-(4-methylphenyl)butanoyl)-D-lysine amide ((*R*)-2c)**

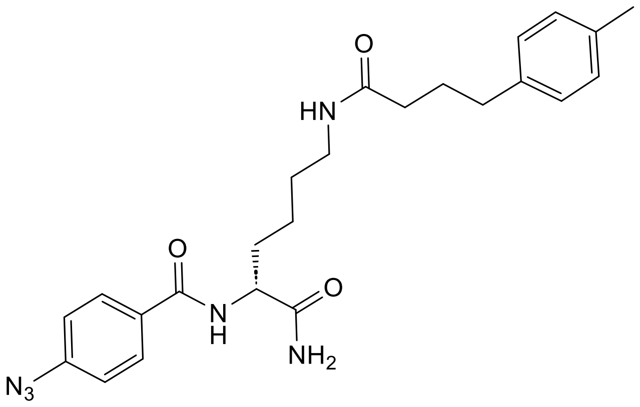

H_25-75_5iso_25min_1mL_42total t_R= 23.5 min

Compound **(*R*)-2c** (99 mg, 28% overall yield, slightly yellow solid) was synthesized starting from Fmoc-D-Lys(Boc)-OH according to the optimized synthetic strategy for carboxamides of lysine-based albumin binders (Scheme 3 in ref [1]. **^{1}H-NMR** (DMSO-d_6) δ = 8.32 (d, ^3J=8.0 Hz, 1H, $N_{\alpha}H$), 7.90 (d, ^3J=7.0 Hz, 2H, H-2,6 azidobenzoyl), 7.73–7.65 (m, 1H, $N_{\varepsilon}H$), 7.33 (s, 1H, N*H*H), 7.14 (d, ^3J=7.0 Hz, 2H, H-3,5 azidobenzoyl), 7.00 (pseudo q, *J*=7.9 Hz, 4H, H-2,3,5,6 tolyl), 6.93 (s, 1H, NH*H*), 4.29 (pseudo q, *J*=7.6 Hz, 1H, $C_{\alpha}H$ lysine), 2.96 (pseudo q, *J*=6.7 Hz, 2H, $C_{\varepsilon}H_2$ lysine), 2.42* (t, ^3J=7.7 Hz, 2H, $C_{\gamma}H_2$ butanoyl), 2.20 (s, 3H, CH_3), 1.97 (t, ^3J=7.5 Hz, 2H, $C_{\alpha}H_2$ butanoyl), 1.73–1.60 (m, 4H, $C_{\beta}H_2$ lysine and $C_{\beta}H_2$ butanoyl), 1.40–1.18 (m, 4H, $C_{\delta}H_2$ and $C_{\gamma}H_2$ lysine). *This signal overlaps with the solvent signal. **^{13}C-NMR** (DMSO-d_6) δ = 173.91 (C=O), 171.63 (C=O), 165.33 (C=O), 142.25 (C-4 azidobenzoyl), 138.61 (C-1/4 tolyl), 134.53 (C-1/4 tolyl), 130.77 (C-1 azidobenzoyl), 129.43 (C-2,6 azidobenzoyl), 128.81 (2xCH tolyl), 128.16 (2xCH tolyl), 118.72 (C-3,5 azidobenzoyl), 53.25 (C_{α} lysine), 38.19 (C_{ε} lysine), 34.84 (C_{α} butanoyl), 34.24 (C_{γ} butanoyl), 31.25 (C_{β} lysine), 28.90 (C_{y} lysine), 27.15 (C_{β} butanoyl),

23.23 (C_γ lysine), 20.60 (CH_3); MS (ESI^+): m/z calculated for $C_{24}H_{30}N_6NaO_3$: 473.23 $[M+Na]^+$, found: 473.19.

TATE derivatives

NODAGA-cLAB5-TATE

NODAGA-cLAB5-TATE (4.7 mg, 14%) was obtained as a white solid.

HPLC chromatogram

H_25-75_5iso_25min_1mL_42total

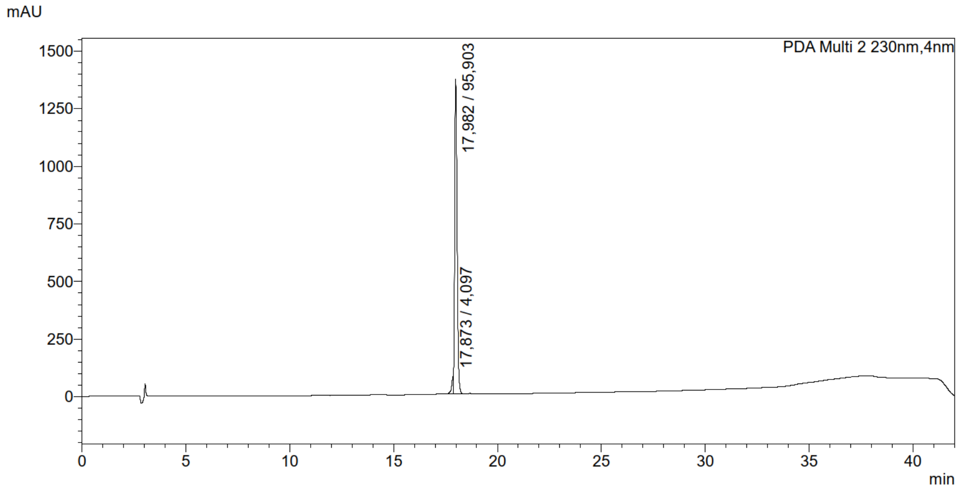

PDA Ch2 230nm

Peak#	Ret. Time	Area	Height	Conc.	Unit	Area%
1	17,873	461295	80115	0,000		4,097
2	17,982	10797713	1366312	0,000		95,903
Total		11259007	1446427			100,000

HRMS spectrum

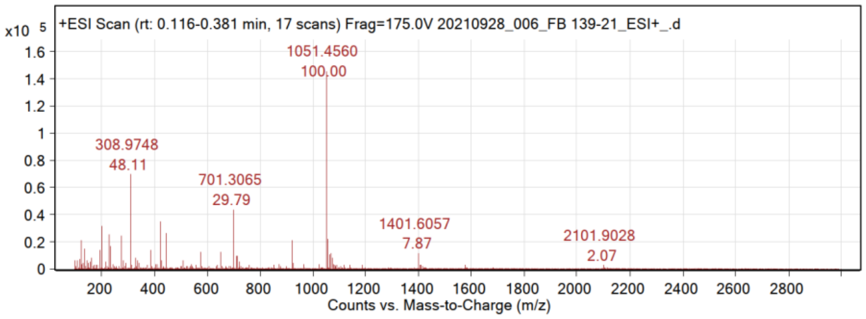

Peak List

m/z	z	Abund
203.0812	1	31635.92
308.9748	1	70012.27
424.0695	1	34754.61
700.9721	1	38396.63
701.3065	1	43348.15
701.6405		31125.08
1050.9543	2	120984.27
1051.456	2	145524.09
1051.9566	2	99921.2
1052.4573	2	51759.61

NODAGA-cLAB6-TATE

NODAGA-cLAB6-TATE (4.4 mg, 6%) was obtained as a white solid.

HPLC chromatogram

H_25-75_5iso_25min_1mL_42total

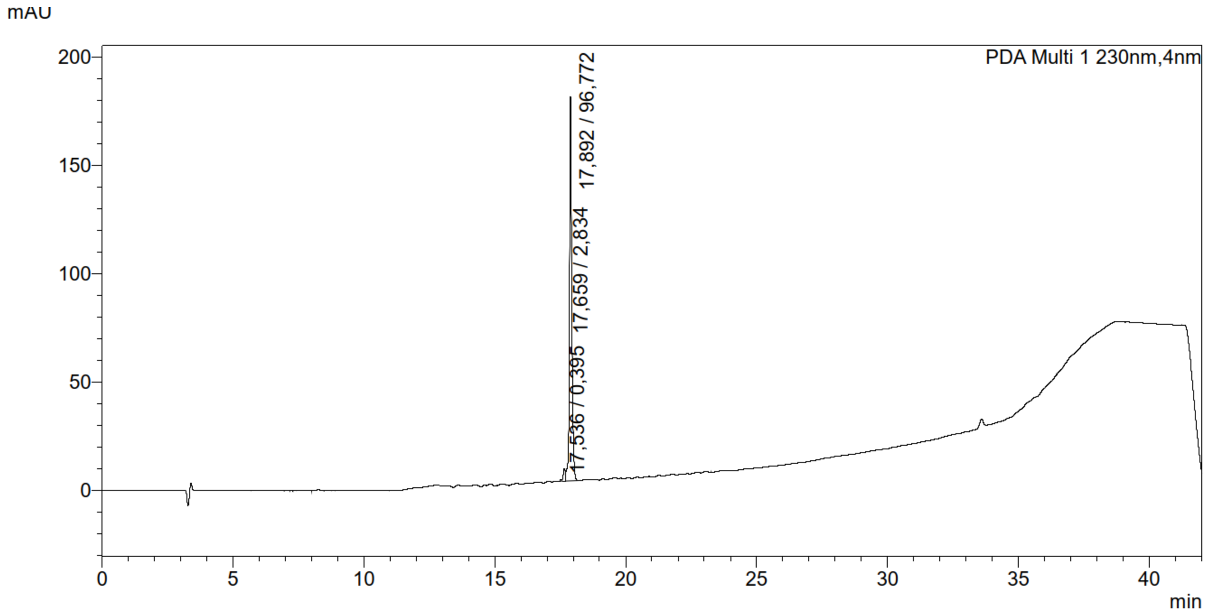

PDA Ch1 230nm

Peak#	Ret. Time	Area	Height	Conc.	Unit	Area%
1	17,536	4488	958	0,000		0,395
2	17,659	32222	5824	0,000		2,834
3	17,892	1100472	176764	0,000		96,772
Total		1137182	183546			100,000

HRMS spectrum

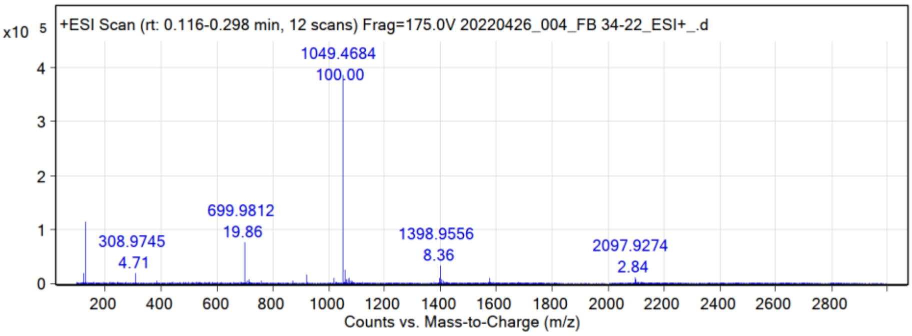

Peak List

m/z	z	Abund
130.1589	1	114118.6
699.6469	1	62785.54
699.9812	1	76912.07
700.3151	1	52683.09
1048.9669	2	307148.66
1049.4684	2	387215.88
1049.9694	2	270407.66
1050.4701	2	138322.91
1050.9705	2	58926.95
1398.9556	1	32371.26

NODAGA-cLAB4-*scr*TATE

NODAGA-cLAB4-*scr*TATE (3.1 mg, 9%) was obtained as a white solid.

HPLC chromatogram

H_25-75_5iso_25min_1mL_42total

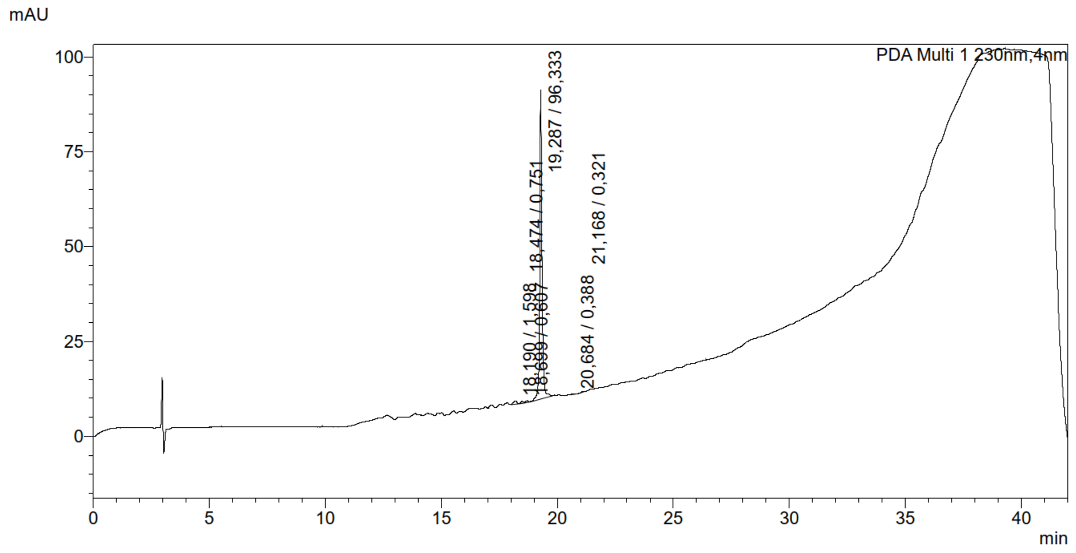

PDA Ch1 230nm

Peak#	Ret. Time	Area	Height	Conc.	Unit	Area%
1	18,190	8976	818	0,000		1,598
2	18,474	4220	507	0,000		0,751
3	18,699	3411	436	0,000		0,607
4	19,287	541032	81230	0,000		96,333
5	20,684	2182	248	0,000		0,388
6	21,168	1804	88	0,000		0,321
Total		561625	83327			100,000

HRMS spectrum

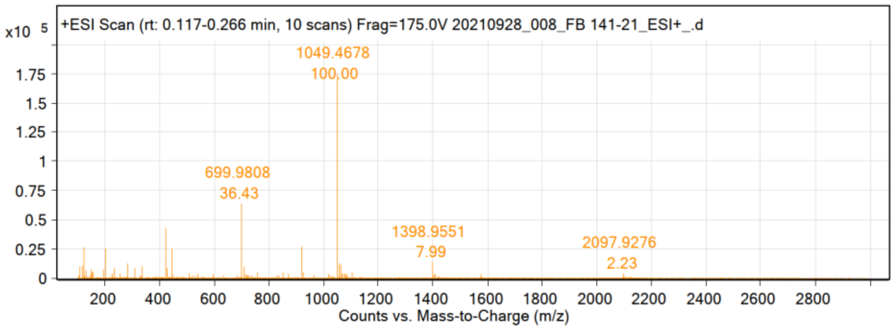

Peak List

m/z	z	Abund
424.0694	1	42853.92
699.6467	1	50544.53
699.9808	1	63673.88
700.3153	1	43330.79
922.0098	1	26889
1048.9662	2	147439.59
1049.4678	2	174807.08
1049.9686	2	121749.91
1050.4693	2	63995.43
1050.9697	2	27975.29

^{64}Cu-labeled TATE derivatives

Exemplary radioactivity-detected chromatograms are shown below after labeling of the different TATE derivatives with copper-64. Labeling yields and purity were ≥98%.

[^{64}Cu]Cu-NODAGA-cLAB5-TATE

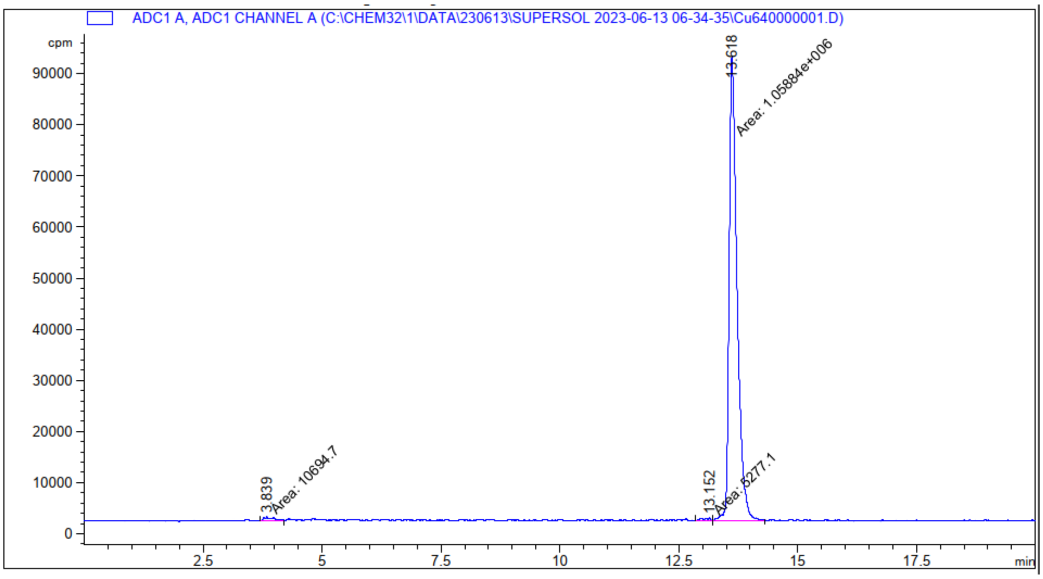

Peak #	RetTime [min]	Type	Width [min]	Area [cpm*s]	Height [cpm]	Area %
1	3.839	MM	0.2201	1.06947e4	809.82513	0.9950
2	13.152	MF	0.1768	5277.10303	497.55286	0.4910
3	13.618	FM	0.1946	1.05884e6	9.06650e4	98.5140

[^{64}Cu]Cu-NODAGA-cLAB6-TATE

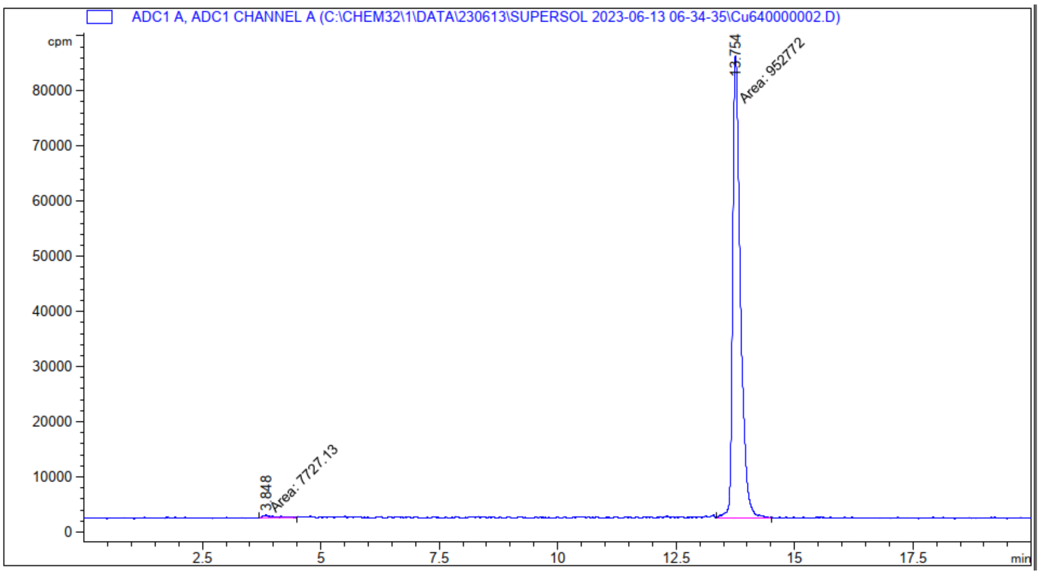

Peak #	RetTime [min]	Type	Width [min]	Area [cpm*s]	Height [cpm]	Area %
1	3.848	MM	0.2583	7727.12549	498.59341	0.8045
2	13.754	MM	0.1896	9.52772e5	8.37375e4	99.1955

[^{64}Cu]Cu-NODAGA-cLAB4-*scr*TATE

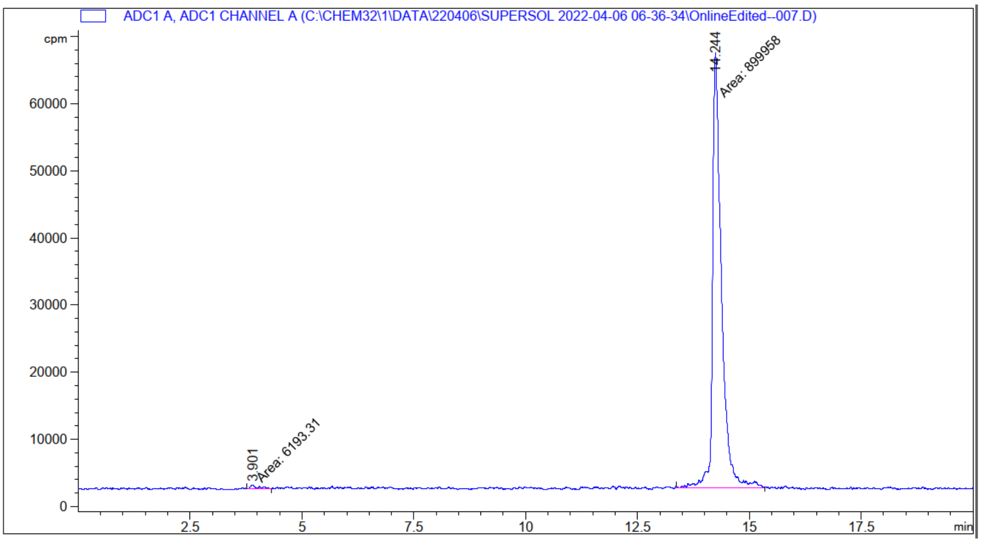

Peak #	RetTime [min]	Type	Width [min]	Area [cpm*s]	Height [cpm]	Area %
1	3.901	MM	0.1909	6193.30518	540.71307	0.6835
2	14.244	MM	0.2314	8.99958e5	6.48219e4	99.3165

[^{64}Cu]Cu-DOTA-EB-TATE

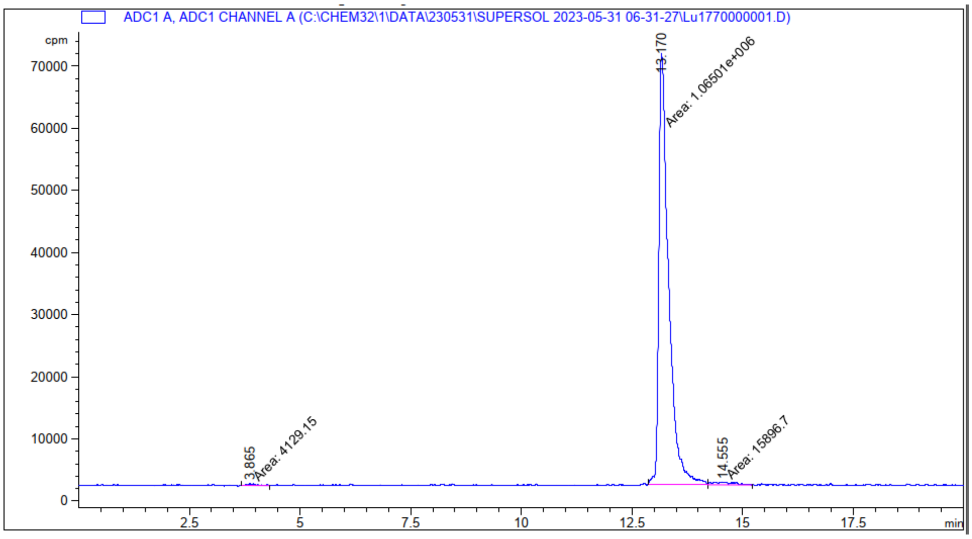

Peak #	RetTime [min]	Type	Width [min]	Area [cpm*s]	Height [cpm]	Area %
1	3.865	MM	0.1990	4129.15479	345.79611	0.3806
2	13.170	MF	0.2557	1.06501e6	6.94200e4	98.1544
3	14.555	FM	0.5468	1.58967e4	484.50357	1.4651

References

1. Brandt, F.; Ullrich, M.; Laube, M.; Kopka, K.; Bachmann, M.; Löser, R.; Pietzsch, J.; Pietzsch, H. J.; van den Hoff, J. and Wodtke, R. "Clickable" albumin binders for modulating the tumor uptake of targeted radiopharmaceuticals. *J. Med. Chem.* **2022**, *65*, 710-733.
2. Ullrich, M.; Wodtke, R.; Brandt, F.; Freudenberg, R.; Kotzerke, J.; Richter, S.; Kopka, K. and Pietzsch, J. The heterobivalent (SSTR2/albumin) radioligand [^{67}Cu]Cu-NODAGA-cLAB4-TATE enables efficient somatostatin receptor radionuclide theranostics. *Theranostics* **2024**, *14*, 5371-5387.
3. Blakeley, D.; Sykes, D. A.; Ensor, P.; Bertran, E.; Aston, P. J. and Charlton, S. J. Simulating the influence of plasma protein on measured receptor affinity in biochemical assays reveals the utility of Schild analysis for estimating compound affinity for plasma proteins. *Br. J. Pharmacol.* **2015**, *172*, 5037-5049.
